# Personalized and graph genomes reveal missing signal in epigenomic data

**DOI:** 10.1186/s13059-020-02038-8

**Published:** 2020-05-25

**Authors:** Cristian Groza, Tony Kwan, Nicole Soranzo, Tomi Pastinen, Guillaume Bourque

**Affiliations:** 1 Human Genetics, McGill University, Montreal, QC Canada; 2 McGill University and Genome Quebec Innovation Centre, McGill University, Montreal, QC Canada; 3 Department of Human Genetics, The Wellcome Trust Sanger Institute, Wellcome Trust Genome Campus, Hinxton, Cambridge, UK; 4 Department of Haematology, University of Cambridge, Cambridge Biomedical Campus, Long Road, Cambdridge, UK; 5 British Heart Foundation Centre of Excellence, Division of Cardiovascular Medicine, Addenbrooke’s Hospital, Hills Road, Cambdridge, UK; 6 The National Institute for Health Research Blood and Transplant Unit (NIHR BTRU) in Donor Health and Genomics, University of Cambridge, Strangeways Research Laboratory, Wort’s Causeway, Cambdridge, UK; 7 Center for Pediatric Genomic Medicine, Kansas City, MO USA; 8 Canadian Centre for Computational Genomics, Montreal, QC Canada; 9 Institute for the Advanced Study of Human Biology, Kyoto University, Kyoto, Japan

**Keywords:** Personalized genomes, Genome graphs, De novo assembly, Modified reference, Reference bias, ChIP-seq, Epigenomics

## Abstract

**Background:**

Epigenomic studies that use next generation sequencing experiments typically rely on the alignment of reads to a reference sequence. However, because of genetic diversity and the diploid nature of the human genome, we hypothesize that using a generic reference could lead to incorrectly mapped reads and bias downstream results.

**Results:**

We show that accounting for genetic variation using a modified reference genome or a de novo assembled genome can alter histone H3K4me1 and H3K27ac ChIP-seq peak calls either by creating new personal peaks or by the loss of reference peaks. Using permissive cutoffs, modified reference genomes are found to alter approximately 1% of peak calls while de novo assembled genomes alter up to 5% of peaks. We also show statistically significant differences in the amount of reads observed in regions associated with the new, altered, and unchanged peaks. We report that short insertions and deletions (indels), followed by single nucleotide variants (SNVs), have the highest probability of modifying peak calls. We show that using a graph personalized genome represents a reasonable compromise between modified reference genomes and de novo assembled genomes. We demonstrate that altered peaks have a genomic distribution typical of other peaks.

**Conclusions:**

Analyzing epigenomic datasets with personalized and graph genomes allows the recovery of new peaks enriched for indels and SNVs. These altered peaks are more likely to differ between individuals and, as such, could be relevant in the study of various human phenotypes.

## Background

**Fig. 1:**
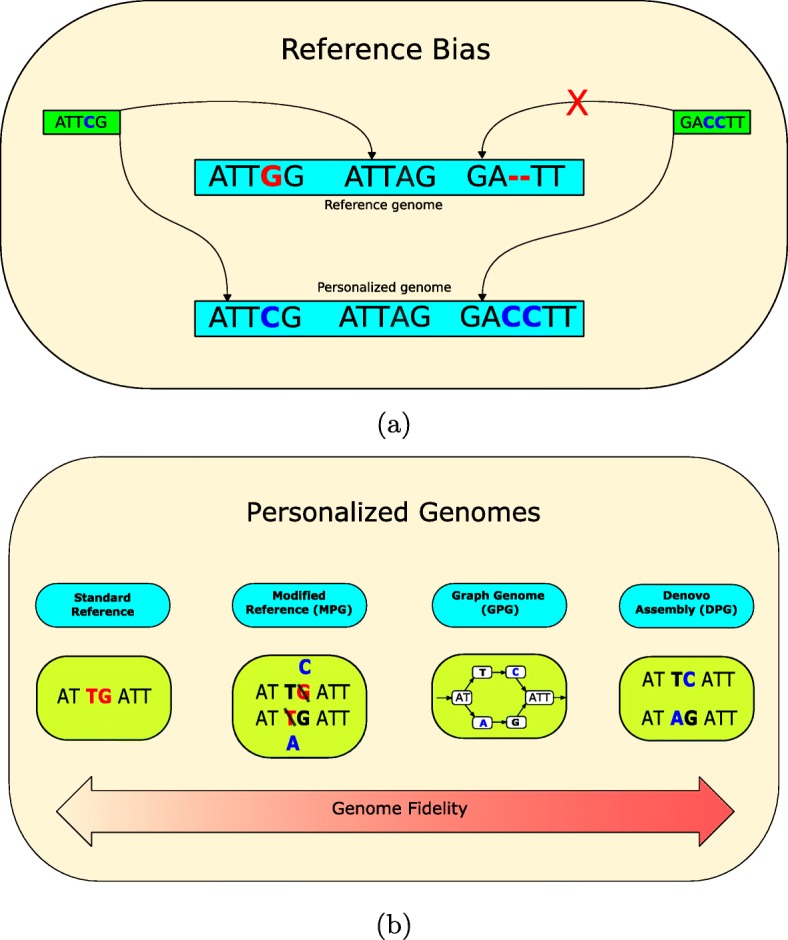
**a** Two instances of reference bias that could be corrected by a personalized genome. One read is mapped to the incorrect location in the reference genome. The other read is unmapped in the reference genome, but becomes mapped in the personalized genome. **b** Phased personalized genomes can be implemented in several ways. The reference can be patched with called variants to create a pair of modified personal genomes (MPGs). Alternatively, a sequence graph genome could be augmented with an individual’s alleles (GPG). Finally, the entire personal genomic sequence can be assembled de novo (DPG)

Standard ChIP-seq analysis relies on aligning reads to a reference sequence followed by peak calling [1, 2]. While the reference genome is a good approximation of the sequence under study, it does not account for the millions of small genetic variants, the larger structural variants, or the two haplotypes of the human genome [3]. Instead, aligners cope with variation by allowing mismatches and indels in read alignments [4]. For example, reads that align to the SNP shown in Fig. 1a would simply include a mismatch in their alignment to the reference sequence. Differences between the genome under study and the reference will shift the mapping of some reads and generate unmapped reads (Fig. 1a), a phenomenon known as reference bias [5]. Provided that the mapping of a number of reads is modified, an alignment to a personalized genome could lead to the gain or the loss of a peak, or what we will call an altered peak (AP). Actually, it has already been shown that just changing the assembly version of the reference can affect epigenomic analyses [6].

In the current study, we want to evaluate the impact of using different types of personalized genomes on ChIP-seq analysis (Fig. 1b). One obvious way of generating a personalized genome is to modify the reference genome using phased variant calls obtained from whole-genome sequencing to generate a diploid pair of sequences [7]. We call this making a modified personalized genome (MPG). Because we cannot align reads to both MPGs simultaneously, analyses are done separately for each haploid sequence and merged afterwards. The advantage is that aligned reads would no longer feature the mismatch corresponding to the SNP mentioned above (Fig. 1b). Epigenomic studies involving the use of MPGs are present in the literature. For instance, Shi et al. modified the reference genome using phased single nucleotide variant (SNV) calls and then realigned transcription factor and histone ChIP-seq data to record allelic specific binding events [8]. However, that study did not consider indels and was limited to understanding how SNVs affect standard analyses but not the identification of APs. Additionally, although pipelines such as AlleleSeq [7] do support indels and structural variations (SVs), they remain restricted to detecting allellic specific events without providing a way to detect APs. Allim [9] is a similar pipeline that attempts to detect instances of allelic imbalance in gene expression by modifying the reference to construct parental haplotypes. Turro et al. also leveraged genotypes, this time by modifying a reference transcriptome [10]. A study that did look at the use of MPGs as compared to the reference genome was done in the context of RNA-seq [11], where it was shown that personalized mouse genomes can improve transcript abundance estimates.

Improving the reference using SNVs and indels can help account for variation of small length, but not for larger SVs. For this reason, we also turn to de novo assembled personal genomes (DPGs) to fully reconstruct the genome sequence under study and to capture a broader range of genetic differences (Fig. 1b). Here, we employ a phased de novo assembly of NA12878. Like MPGs, it provides a sequence for each haploid but it is not constructed from the reference genome. However, high-quality DPGs remain challenging to obtain for epigenomic analyses, as they typically require at least 50× sequencing depth and long reads, which remain costly [12]. Also, the computational time for DPGs is much higher than aligning to a reference and calling variants [13]. Moreover, de novo assemblies may contain defects and are often incomplete compared to the reference [14]. Despite this, they may still provide a useful point of comparison.

Finally, the above trade-offs also motivate the exploration of graph genomes as an additional strategy. Graph genomes are a flexible way of representing many possible sequences in a concise data structure [15]. Unlike traditional one-dimensional sequence representations, graph genomes split sequences into segments called nodes. The nodes are connected to each other by edges, which allows traversing the graph from one node to another. Well-defined rules about the semantics of nodes and the direction of edges allow graphs to express many sequences. A valid traversal is called a path and represents one possible sequence that is represented by the graph. This emerging technology can encode sequence variation at many levels for different purposes [16]. For example, it can encode genetic variation within a population of the same species, genomic differences between species within a phylogenetic tree, or genomic rearrangements of a cancerous tumor. We will employ graph genomes to construct a graph personalized genome (GPG) representing the diploid genome of a single individual (Fig. 1b). GPGs can leverage available call sets that include a broad range of variants, from SNPs and indels to catalogs of sequence resolved SVs, and also capture the diploid nature of the human genome [17]. This is achieved by converting the reference genome to a graph format and augmenting it with nodes representing the variants. By mapping to a GPG, we expect that reads containing variants will align to the appropriate path, which improves read alignment accuracy [5]. Conveniently, genome graph implementations such as vg [18] exist and provide the proper utilities and semantics to work with annotations spanning multiple coordinate systems. Moreover, there are tools that can call ChIP-seq peaks directly from graph genomes [19].

The objective of our study is to provide a comparison between alternative personalized genomes (MPGs, DPGs, and GPGs) for ChIP-seq analyses. We focus on the H3K4me1 and H3K27ac histone marks primarily due to broad availability in samples of the Blueprint Consortium [20] (see below). H3K27ac is linked with enhancers, distinguishing between active and inactive enhancers and therefore impacts gene regulation [21]. At the same time, H3K4me1 correlates with H3K27ac in enhancers, but can interact with chromatin regulators such as p300 and other histone marks to determine other classes of regulatory elements [22]. Even if only a fraction of peaks are observed to be altered, these regions will correspond to biochemically active regions that are more likely to differ between individuals and, as such, could be relevant in the study of various human phenotypes.

## Results

### Modified personal genomes alter a small fraction of peaks that are enriched in indels

There are many high-confidence variant call sets and assemblies of the NA12878 genome, which makes it a good candidate for benchmarking [23, 24]. We created a paternal and maternal MPG for NA12878 and aligned whole-genome sequencing (WGS) reads to the standard human reference and to these MPGs (see the “Methods” section). We wanted to estimate the proportion of changed mappings and noted that 3.6% of WGS reads move depending on the reference that is used (Additional file 1: Table S1a). To measure the impact of reads changing location on ChIP-seq calls, we aligned H3K4me1 and H3K27ac ENCODE datasets from NA12878 and counted the proportion of altered peaks (see the “Methods” section). Altered peaks are categorized into two categories. Peaks in the personalized genome that do not overlap a peak in the reference genome are called personal-only. Peaks in the reference genome that do not overlap a peak in the personalized genome are called ref-only. Any peak in the personal genome that overlaps a peak in the reference genome is a common peak (see the “Methods” section). We found that the fraction of personal-only and ref-only peaks was consistent between the two histone marks (Table 1). Among the H3K4me1 calls, each MPG yielded roughly 1600 personal-only (1.1%) peaks and roughly 800 ref-only peaks (0.6%). Among the H3K27ac calls, we called roughly 600 personal-only peaks (1.0%) and 300 ref-only peaks (0.5%) in each MPG. Notably, personal-only peaks were found at about double the rate of ref-only peaks. Ref-only peaks arise when the reads forming a peak pileup in the reference map to different locations in the personalized genome. In contrast, personal-only peaks emerge when reads shift their mapping from the reference pileup to the new personalized pileup or when reads that did not map to the reference become mapped to the personalized genome. Consistent with this hypothesis, there was a net gain of mapped WGS reads in the NA12878 MPG (Additional file 1: Table S1a) and personal-only intervals are enriched in ChIP-seq rescued reads relative to ref-only intervals (Additional file 1: Fig S1a).

**Table 1:** Number of altered peak calls in MPGs, DPGs, and GPGs for the NA12878 H3K4me1 and H3K27ac marks

Version	Mark	Common	Personal-only	Ref-only
MPG, paternal	H3K4me1	146,520	1636 (1.1%)	854 (0.6%)
MPG, maternal	H3K4me1	146,570	1622 (1.1%)	808 (0.6%)
MPG, downsampled	H3K4me1	146,688	1051 (0.7%)	550 (0.4%)
DPG, Hap1	H3K4me1	141,444	7176 (4.8%)	6755 (4.6%)
DPG, Hap2	H3K4me1	141,442	7130 (4.8%)	6774 (4.6%)
DPG, Pendleton	H3K4me1	142,347	16,245 (10.2%)	8912 (5.8%)
GPG	H3K4me1	132,668	3068 (2.3%)	1178 (0.9%)
MPG, paternal	H3K27ac	68,888	660 (1.0%)	351 (0.5%)
MPG, maternal	H3K27ac	68,909	688 (1.0%)	335 (0.5%)
MPG, downsampled	H3K27ac	68,953	438 (0.6%)	218 (0.3%)
DPG, Hap1	H3K27ac	63,419	2078 (3.2%)	9901 (13.5%)
DPG, Hap2	H3K27ac	63,441	2091 (3.2%)	9899 (13.5%)
DPG, Pendleton	H3K27ac	66,811	5208 (7.2%)	4980 (6.9%)
GPG	H3K27ac	75,538	1847 (2.4%)	1206 (1.6%)

**Fig. 2:**
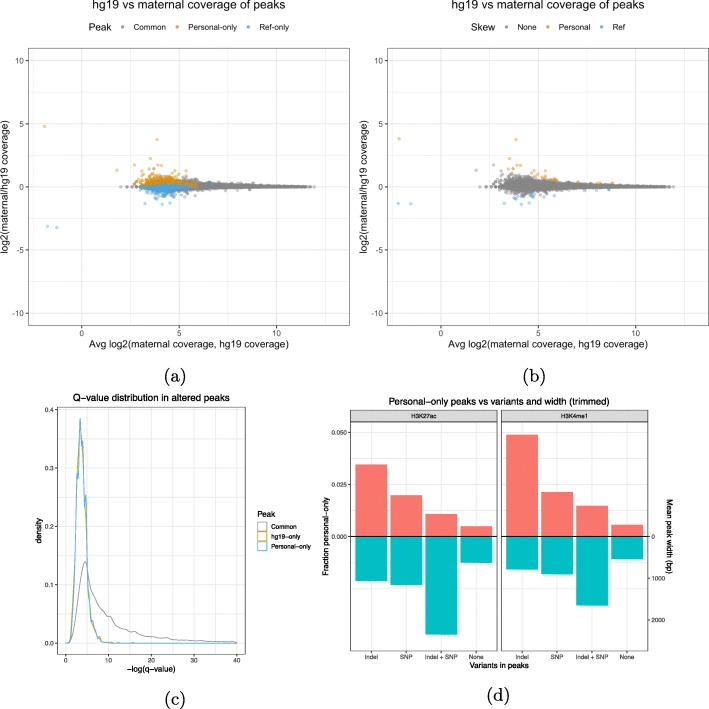
**a** A comparison of the coverage of H3K4me1 peak called regions in hg19 and the maternal MPG. **b** Identification of peak called regions that have a significant difference in coverage. **c***Q* value distributions of the same H3K4me1 peaks. **d** NA12878 MPG estimate of the probability that each combination of variation calls present in a region may cause a personal-only peak call compared to their average widths

Aligning to a personalized genome may cause differences in read density that do not necessarily lead to an AP call, especially in the strong peak regions. For that reason, we also counted the reads in personal-only, ref-only, and common peak intervals and compared them between the reference and personalized alignments (see the “Methods” section). Ideally, AP calls should also have a skewed coverage toward the personal or reference genome that indicates a clear change in read depth at that site. However, for most AP calls, we found that their coverage distribution remained clustered within the distribution of the common and unaffected peak calls (Fig. 2a). Most affected peak calls fall into the no-skew category together with common calls, with only around 30 out of 1600 peaks having a coverage skewed toward the reference or the MPG (Fig. 2b and Table S2). Comparing the *q* value distribution of common peaks against the distribution of APs revealed similar modes but a much shorter right tail for APs (Fig. 2c). This means that personal-only peaks and ref-only peaks are confined to a region of narrower width and lower confidence (as measured by MACS2 score) than most common peaks (Additional file 1: Fig S1b - S1c). Similar results were also observed for H3K27ac (Additional file 1: Fig S2a and S3a).

Finally, we wanted to explore the link between AP and variant calls, as we expected the former to occur mainly in the presence of the latter. For this purpose, we binned AP calls according to the overlapped combination of variants (see the “Methods” section). Reassuringly, we found that peak calls that do not contain variations have a near zero chance of being altered, while peaks overlapping at least one indel are the most likely to be altered followed by peaks overlapping at least one SNP (Fig. 2d). Interestingly, peaks containing at least one SNP and indel are the least likely to be altered. A factor that could explain this trend is the peak width associated to each peak category and histone mark. Indeed, we found that the mean width of peaks overlapping both indels and SNPs is the highest among the four combinations of variations, followed by peaks with at least one indel and peaks with at least one SNP (Fig. 2d). Using a regularized logistic regression model (see the “Methods” section), we were also able to show that peak width has an inverse relationship with AP calls (Additional file 1: Fig S1d - S1e). We estimated that the AP call log-odds ratio decreases by 0.19 per additional 100 bp in peak width and increases by 1.29 per additional SNP and by 2.0 per additional indel. This model predicts fewer altered peaks in broad histone marks and more altered peaks in narrow histone marks and transcription factors.

### Applying modified personal genomes to Blueprint samples

**Fig. 3:**
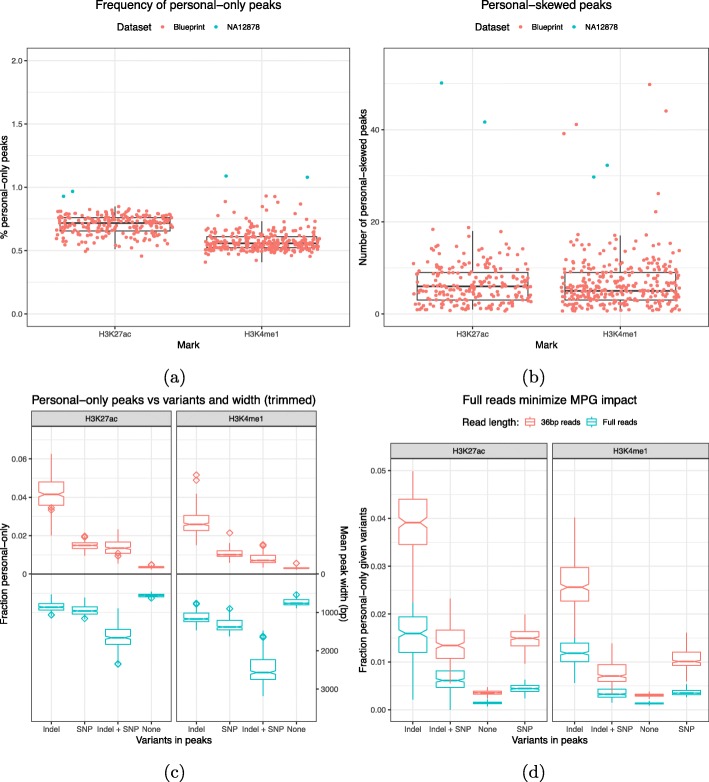
**a** Proportion of peaks that are called only in personalized MPGs. **b** Number of peaks with higher coverage in the personalized MPG than in the reference. **c** Blueprint MPG estimates of the probability that each combination of variation calls present in a region may cause a personal-only peak call compared to their relative average widths. **d** The probability that a variant affects a peak called on full reads is lower compared to trimmed reads

NA12878 is a deeply sequenced sample with high-quality variant calls, meaning that it is not representative of most datasets. We wanted to evaluate the proportion of altered peaks on lower pass WGS datasets such as Blueprint, a cohort of samples used in the study of hematopoietic epigenomes for which ChIP-seq data is available [20] (see the “Methods” section). In Blueprint samples, we called on average 130 and 47 thousand common peaks for H3K4me1 and H3K27ac, respectively. Overall, the total number of peaks is comparable to NA12878 (Additional file 1: Table S3). In H3K4me1, there are approximately 750 (0.6%) personal-only peaks and 450 (0.4%) ref-only peaks. In H3K27ac, there are approximately 330 (0.7%) personal-only peaks and 190 (0.4%) ref-only peaks. Among these samples, the number of APs is almost always below the NA12878 benchmark (Fig. 3a and S4a). Again, ref-only peaks are observed to occur less often than personal-only peaks. A decrease is also observed with skewed peaks. While not numerous in the benchmark to begin with (50–70), their number in the typical Blueprint sample barely reaches double-digit numbers (Fig. 3b and S4b). This is likely due to the difference in the whole-genome sequencing depth, as the NA12878 variant call set (3.5M SNPs, 0.5M indels) is richer than Blueprint (approximately 3.25M SNPs and 0.375M indels per sample, Additional file 1: Fig S4c). We confirmed this by creating a NA12878 MPG by downsampling the original set to 2.6M SNVs and 100K indels. As shown in Table 1, the downsampled MPG produces fewer AP calls relative to the full set for both H3K4me1 and H3K27ac marks. We should also keep in mind that the phasing of NA12878 variant calls is better than for Blueprint, which could also contribute to more AP calls.

In Blueprint, altered peaks remain enriched in variants, with peaks containing indels being altered most frequently (Fig. 3c). Again, we found that the peaks of H3K4me1 were slightly less likely to be altered than the peaks of H3K27ac. As previously discussed, this is probably due to the inverse relationship between peak width and altered calls. As to the quality of altered Blueprint peaks, the same pattern of width, confidence, and coverage observed in NA12878 was seen again in Blueprint samples (Additional file 1: Fig S5). The small differences in coverage together with the weak confidence of APs indicate that MPGs can only alter the calls of regions that are very near the threshold of significance.

Finally, in this initial analysis, we had trimmed every sample to a read length of 36 bp to make it comparable to the NA12878 datasets (see the “Methods” section). To test the effect of read length, we repeated the Blueprint analysis with the full 100 bp reads. We found, as expected, that as the read length increases, APs become less likely (Fig. 3d). We repeated the NA12878 WGS alignment comparison with the longer 100 bp reads to gain some insight on why this happens (Additional file 1: Table S4a). Compared to the shorter reads (Additional file 1: Table S1a), the longer reads show a small decrease in aligned reads with unequal mappings. However, the proportion of reads are mapped in one genome but not in the other halves. This is accompanied by a greater mapping rate of the whole WGS dataset. Therefore, the decrease in APs can be attributed to a smaller proportion of reads that are rescued by the personalized genome.

### De novo personalized genomes create a larger number of altered peaks

If the moderate effect of using MPGs for ChIP-seq calls in NA12878 and Blueprint is explained by the fact that larger scale variations had not been taken into account, then de novo assemblies, or DPGs, could potentially have a broader impact. Support for this hypothesis comes from the increased rate of read mapping changes when using DPGs instead of MPGs (Additional file 1: Table S1b). We opted to use the 10× Hap1 de novo assembly as a DPG for this comparison (see the “Methods” section). In this DPG, 9.8% of reads change their mapping, which is nearly a threefold increase from the equivalent analysis with MPGs. When using full reads, we still get that 9.4% of reads alter their mapping (Additional file 1: Table S4b). As in MPGs, the number of rescued reads proportionally changes the most.

**Fig. 4:**
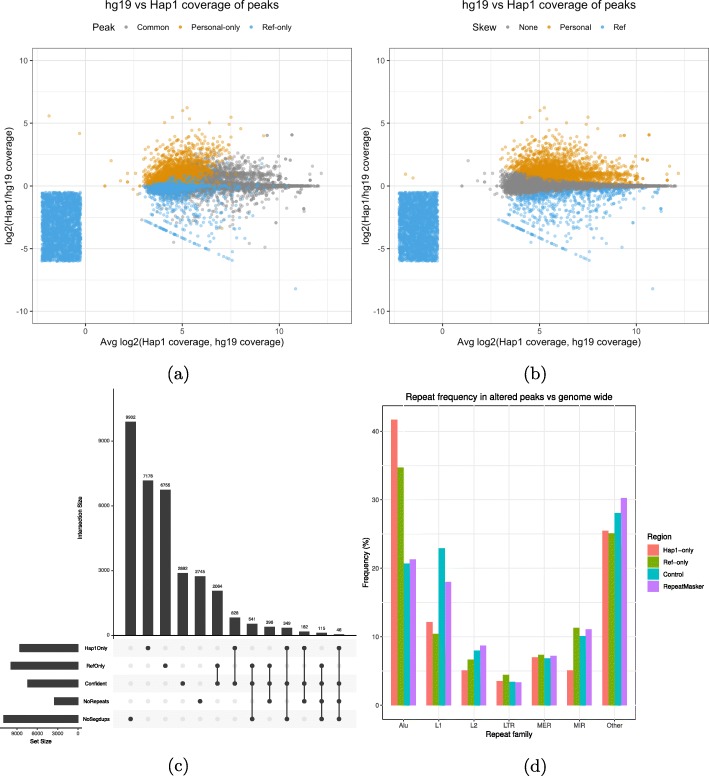
**a** A comparison of the coverage of peak called regions in the reference and the Hap1 DPG. The smear represents ref-only peaks with no coverage in Hap1. **b** Identification of peak called regions that have a significant difference in coverage. **c** Summary of the overlap between altered peaks, confident peaks, repeats, and segmental duplications [58]. **d** The repeats that overlap altered peaks are enriched in Alu elements relative to their frequency in the RepeatMasker. The categories are chosen by grouping repeats by name prefix, summing their frequencies per group, and taking the largest groups. Remaining groups are labeled as “other.” The control regions are random genomic intervals with a width distribution identical to altered peaks

In the context of ChIP-seq analysis, this should lead to a larger number of altered peaks. Indeed, using the same datasets (see the “Methods” section), we found that the altered peak calls are roughly five times more numerous with a similar number of common peaks when using the Hap1 and Hap2 DPGs instead of an MPG (Table 1). For H3K4me1, we obtained approximately 7.1 thousand (4.8%) personal-only peaks and 6.7 thousand (4.6%) ref-only peaks. For H3K27ac, we called approximately 2.1 thousand (3.2%) personal-only peaks. For this mark, the number of ref-only peaks is unusually large at 9.9 thousand (13.5%) peaks. We also repeated the analysis that identifies peaks that have skewed read counts toward the DPG or the reference. Notably, we found that many AP calls now have substantial differences in coverage (Fig. 4a and Additional file 1: Fig S2c for H3K27ac). There are also many significantly skewed peaks, with a larger read count difference between the reference and the DPG (Fig. 4b and Additional file 1: Fig S2d for H3K27ac). Similar results are also obtained using the Pendleton DPG (see the “Methods” section and Table 1). Overall, personal-skewed and ref-skewed peaks are one to two orders of magnitude more numerous in DPGs versus MPGs (Additional file 1: Table S2). Although personal-only peaks do not reach an identical distribution to common peaks, there are considerable gains in terms of width and quality (Additional file 1: Fig S6a). DPG-only peaks are found to have a higher mean SNP and indel density compared to common peaks (Additional file 1: Fig S6d). As for ref-only peaks, they are only slightly enriched in variation calls. This can be explained by a group of ref-only calls that have coverage in the reference but not in the DPG (Fig. 4a). We view these ref-only peaks as probably missing from the de novo assembly and not as the product of genetic variation.

If SVs are the root of many AP calls, then many of these peaks should overlap repeats or segmental duplications that are known to be underrepresented in de novo assemblies [25]. We selected the most confident subset of H3K4me1 AP calls to be overlapped with segmental duplication (SD) and repeat annotations (see the “Methods” section). This reduces the initial set to 828 confident DPG-only and 2064 confident ref-only peaks. Among confident DPG-only peaks, only 349 peaks are located in regions free of SDs (Fig. 4c). Ref-only peaks with positive DPG coverage register much fewer SDs (6.3%). However, ref-only peaks without DPG coverage are highly associated with SDs (71.3%) (Additional file 1: Table S5). The lack of coverage suggests that these duplicated sequences are not present in the DPG. Looking among the SD-free peaks, we discovered peaks with large differences between the reference alignment and the DPG alignment (Additional file 1: Fig S7). We also measured the enrichment in APs of the different repeat families (see the “Methods” section). Alus were found to be 2 times more frequent in DPG-only peaks and 1.5 times in ref-only peaks (Fig. 4d). The same is not true for repeat families such as L1, which occur equally or less often in APs relative to the genome. There also exists a small confident subset of 46 DPG-only and 115 ref-only peaks that are free of both SDs and repeats. Despite the absence of known repeats or segmental duplications, these peaks can still have large differences in coverage between the DPG and the reference alignments (Additional file 1: Fig S8). We obtained similar results for H3K27ac (Additional file 1: Fig S9a - S9b).

### Graph personalized genomes create more altered peaks than MPGs

Although DPGs are more effective than MPGs to recover APs, in practice, they are often difficult to obtain. Therefore, we were interested in GPGs due to their ability to represent genetic variation and potentially approximate de novo assemblies by exploiting structural variant catalogs. In addition, GPGs improve on MPGs by allowing read alignment to a diploid genome instead of treating each haploid individually. As before, we mapped the same WGS reads to the reference genome, this time represented as a graph, and to the NA12878 GPG and then compared their coordinates using built-in vg functionality (see the “Methods” section). By properly representing the diploid genome, we expected GPGs to shift the mapping of a greater proportion of reads than an equivalent pair of MPGs. In fact, we found that the proportion of unequal mappings between the reference graph and the NA12878 GPG (8.3%) is more than twice the number between the reference and the NA12878 MPGs (3.43%) given the same WGS dataset (Additional file 1: Table S1c). We verified that this proportion remains stable when varying alignment mismatch and gap penalties (Additional file 1: Fig S10a).

**Fig. 5:**
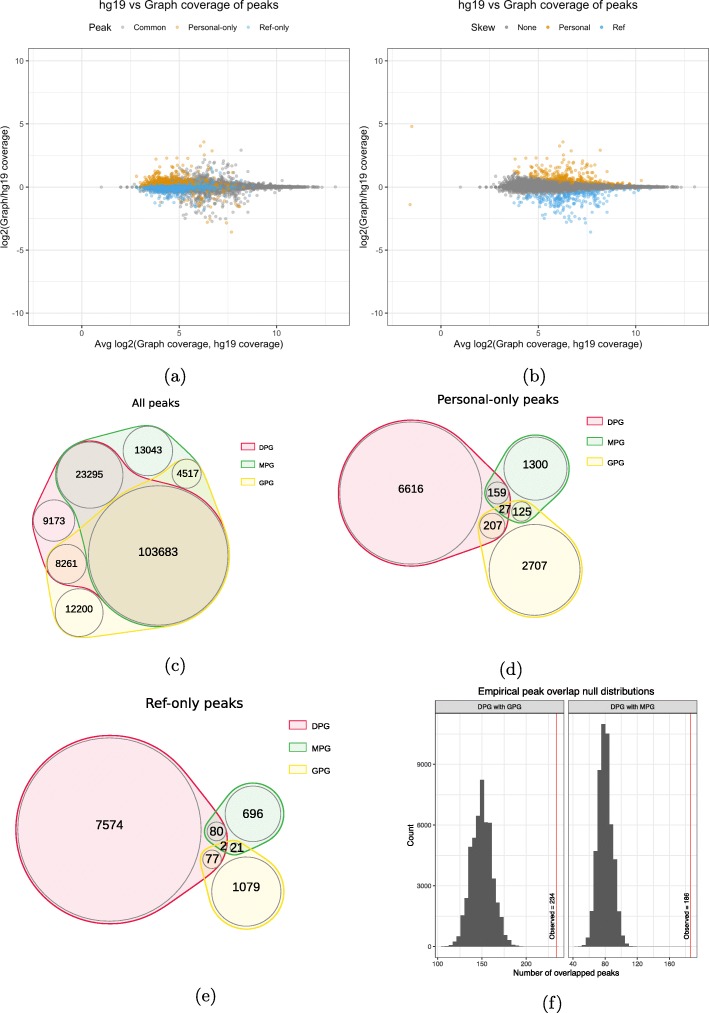
**a** A comparison of the coverage of H3K4me1 peak called regions in the reference and the graph genome. Pairwise overlaps between MPG, DPG, and GPG H3K4me1 peak tracks. **b** Identification of peak called regions that have a significant difference in coverage. **c** Overlap of all peak calls. **d** Overlap of altered personal-only peak calls. **e** Overlap of ref-only peak calls. **f** Empirical null distributions for the overlap of personal-only peaks between personal genome implementations

We found similar numbers of common peaks in GPGs as in MPGs and DPGs, specifically 132 thousand H3K4me1 calls and 75 thousand H3K27ac calls (see Table 1 and the “Methods” section). Among the H3K4me1 calls, 3068 (2.3%) are personal-only and 1178 (0.9%) are ref-only. Among the H3K27ac calls, 1847 (2.4%) are personal-only and 1206 (1.6%) are ref-only. Both sets of values are intermediate between MPGs and DPGs (Table 1). Revisiting the peak read counts between the reference graph and the diploid graph shows greater dispersion, among both altered and common peaks (Fig. 5a). The same test for read count skew yields between 279 and 411 peaks, an order of magnitude more than MPGs (Fig. 5b, Additional file 1: Table S2). See also Additional file 1: Fig S2e - S2f for similar results with H3K27ac. Next, we recalculated the association of indels and SNPs with the personal-only peak calls in GPGs (Additional file 1: Fig S11b). Again, indels have the strongest association with APs for both H3K4me1 and H3K27ac marks. Contrary to MPGs, H3K27ac peaks containing both indels and SNPs are just as likely to be altered as peaks containing only SNPs, despite being much wider. Similarly to MPGs, peaks lacking variants are the least likely to be altered in both histone markers.

The false discovery rate (FDR) is an important parameter in peak calling. It is possible that a peak that is personal-only at a given FDR would be found in the reference at higher FDR. Testing for this, we found that for H3K27ac, 1021 of 1847 peaks remain personal-only at 0.05 FDR even when using 0.10 FDR in the reference. For H3K4me1, 745 of 3068 peaks remain personal-only. This suggests that personal genomes move some read pileups just above the significance threshold. We know that personalized genomes improve alignment accuracy [5] and that individualized genomes improve transcript abundance estimates in RNA-seq [11]. Provided the signal also increases in ChIP-seq, we argue that the peak ranking in personalized genomes is more meaningful than the ranking in the reference genome. The excess of personal-only to ref-only peaks supports the new ranking. If the ranking changed randomly, the number of personal-only peaks would equal ref-only peaks. We also check that the number of personal-only was not too sensitive to the choice of FDR threshold (Additional file 1: Fig S12). We ran even more ENCODE datasets through our GPG pipeline to measure the proportion of APs across the NA12878 reference epigenome (Additional file 1: Fig S13). GPG-only peaks were found to vary from 0.84% for CTCF to 6.53% for H3K9me3.

An additional concern is that the FDR of peak callers such as MACS2 may be inaccurate. Therefore, we tested IDR, a tool that compares the ranking of peaks between replicates to better control the FDR [26]. Using our approach on an additional NA12878 ENCODE dataset (H3K4me3), due to the availability of consistent replicates with similar read depth and read length, we found that the proportion of personal-only peaks in this dataset declines from 0.99 to 0.6% if we correct MACS2 peaks with IDR (Additional file 1: Table S6). This shows that the majority of personal-only peaks remain, even if we apply a more stringent statistical cutoff. We also show that personal-only peaks are supported by orthogonal sources of data in addition to replicates. We do so by correlating the peak calls to the read depth in H3K4me1, H3K27ac, and H3K4me3. In common peaks, the average read depth rises together with the average read depth of correlated histone marks (Additional file 1: Fig S14). In personal-only peaks, we observe a similar pattern together with an increased average read depth compared to ref-only peaks. Moreover, since alignment parameters may also be relevant, we confirm that simply aligning ChIP-seq reads to the reference with different mismatch and gap penalties does not change more reference peak calls than a personalized genome (Additional file 1: Fig S10b, S10c).

Next, we were interested in the concordance between the 3 approaches: MGP, GPG, and DPG (see the “Methods” section). We found the overlap between the total peak tracks to be substantial, with over 100,000 H3K4me1 peak calls overlapping between the three personalized genome implementations (Fig. 5c and S9d for H3K27ac). In contrast, when the AP calls are intersected, a small overlap is observed for personal-only peaks and ref-only peaks (Fig. 5d, e and S9e - S9f for H3K27ac). Only 234 of 3068 (7.6%) of the NA12878 GPG personal-only calls are replicated in the DPG. Similarly, only 79 GPG ref-only calls are replicated from a total of 1178 peaks (9.8%). Comparatively, the replication rates between MPGs and DPGs are slightly higher, despite smaller absolute number of peaks. One hun dred eighty-six of 1622 (14%) personal-only peaks and 82 of 808 (10.1%) ref-only peaks are replicated in the DPG. We wanted to know if chance alone could explain this small overlap of AP calls. We checked this by generating a distribution of peak overlaps by randomly and repeatedly sampling the respective number of personal-only peaks in each genome from its total number of peaks (see the “Methods” section). The expected number of replicated personal-only peaks is 140 peaks between the GPG and DPG and 80 peaks between the MPG and DPG (Fig. 5f). As such, albeit small, the number of replicated peaks cannot be explained by chance alone.

### Further characterizing the altered peaks

**Fig. 6:**
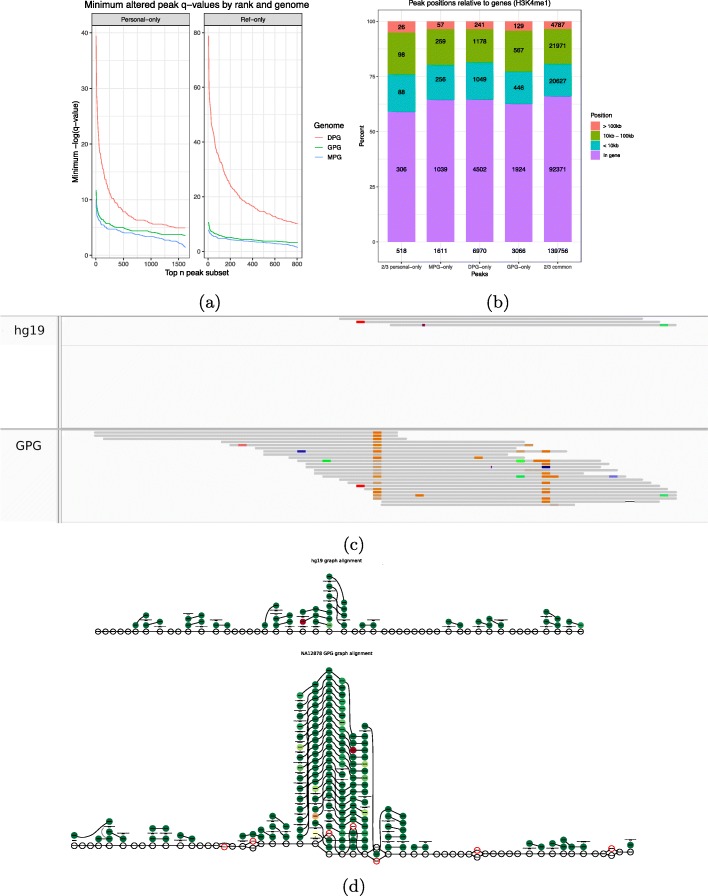
**a** Comparison of altered peak *q* values between MPG, GPG, and DPG implementations by rank. The top *n* peak subset was increased by 5 peak increments. **b** Distribution of gene relative positions of personal-only peaks among all genomes. Personal-only and common peaks replicated in at least two genomes are also featured. **c** The pileup of a GPG-only peak projected to the hg19 linear reference. **d** The true graph rendering of the above AP in the NA12878 GPG and reference genome graph

We were interested in comparing the quality of the APs found by the three different approaches. We did this by comparing the *q* values of peaks by rank in each genome (Fig. 6a). From this, we observed that the best DPG-only peaks surpass the best GPG-only and MPG-only peaks by a wide margin. The top GPG APs only surpass the top MPG APs by around one unit on the − log10(*q*) scale. But on a linear scale, this means that the most confident GPG APs are an order of magnitude more confident than the most confident MPG APs. See Additional file 1: Fig S3c - S3d for H3K27ac.

H3K4me1 is a histone mark known to be associated with gene activation that is present near transcription start sites and transcribed regions [27]. Meanwhile, H3K27 is localized in enhancers [21]. However, these patterns may not necessarily be replicated in AP calls, particularly if they are caused by noisy signal. Therefore, we wanted to check whether APs maintain the same genomic distribution as the rest of the calls, among all three genome implementations. To this end, we computed the distances to the nearest gene for MPG-only, DPG-only, and GPG-only peaks as well as for personal-only and common peaks that were replicated in at least two genomes (see the “Methods” section). We distinguished between peaks that overlap a gene and peaks that are within 10 kb, between 10 kb and 100 kb, or further than 100 kb from a gene. Overall, the genomic profile of AP calls is very similar to that of replicated common calls across the board, regardless of the genome or replication (Fig. 6b and Additional file 1: Fig S9c for H3K27ac).

Given that more than half of APs are within genes, some may be of particular interest. Indeed, Fig. 6c shows a GPG-only example projected to the reference, while Fig. 6d shows the true graph rendering of the pileups. The personalized peak overlaps four consecutive SNVs which are incorporated in the GPG but not the reference graph. Since this peak lies on the alternate allele, future allelic quantification pipelines that operate on graph genomes should be able to detect such events. The graph rendering clearly shows a fair number of reads aligning to these SNVs, forming a pileup that fails to appear in the reference graph. Moreover, this interval is within the third intron of STON1-GTF2A1L, a gene that appears in two GWAS studies linking it to neovascular age-related macular degeneration [28] and polycystic ovary syndrome [29]. Such examples justify investigating whether GPGs could improve our understanding of gene regulation in individual genomes.

## Discussion

By moving from the reference sequence to a MPG, GPG, and DPG, the genome representation became richer by incorporating SNVs and indels, variants in the form of a diploid graph, and also larger structural variants. These personalized genomes provide an upstream benefit by improving read alignment to downstream ChIP-seq peak calling pipelines. When reanalyzing ChIP-seq datasets using these personalized genome implementations, we were able to identify hundreds to thousands of APs using permissive MACS2 and Graph Peak Caller FDR cutoffs. The proportions of altered peaks in GPGs only decreased by a factor of 2 in the stringent IDR analysis that employs ENCODE replicates. While most APs detected using MPGs had only marginal changes in coverage, the GPGs and DPGs yielded tens to thousands of peaks with significant read count differences relative to the reference. Notably, we observed that indels followed by SNVs were enriched in APs and that there was an inverse correlation with peak width. We also observed that Alus were overrepresented in APs, a transposable element known to be active in the human genome [30] and with many polymporphic instances in the population. Although it is tempting to think that some of these APs might be driven by Alu polymorphisms, it would require additional validation as it could also be caused by errors in the personalized genomes that were used for the analysis.

The vast majority of common peaks were identified consistently by the 3 methods, but only a minority of APs were found by 2 or more methods. This limited overlap might be a consequence of the fact that the genome implementations are technically very different from each other. For instance, only DPGs at this stage took into account SVs, but at the same time, some regions of the personal genome might be missing for the current DPG. GPGs represent a promising compromise between MPG and DPGs as they also have the ability to natively account for the diploid nature of the human genome. A natural extension will be to try to incorporate SVs into GPGs to see how it can further improve their performance. Comparing the results of several graph genome aligners that employ different strategies in addition to varying alignment parameters would be interesting once they become widely available. Furthermore, as pangenome graphs are created to capture all known variations [15] together with the haplotypes of entire populations, it might be possible to further improve on the current performance as well as finding associations between genotype and personal-only peaks.

Even though we primarily focused on APs, we also encountered peaks that differed significantly in read counts. These skewed common peaks produced by personalized genomes should not be ignored, particularly when performing differential expression analysis between control and treatment groups. Even if the number of skewed peaks is generally smaller than APs, they remain important because such studies typically identify a small number of differentially expressed regions. Therefore, the application of personalized genomes could reveal new data points or correct false positives.

In studying the impact of using personalized genomes with epigenomic data, we initially focused on the human species because of the high quality of its reference, which allowed us to reliably estimate bias. However, the above results could be amplified in species of greater genetic diversity such as chimps [31]. Notably, to properly determine the biological significance of APs, our analysis will need to be expanded to datasets obtained from more tissues and with follow-up experiments. Finally, we constructed personalized genomes that only incorporate germline genetic variation. However, this does not account for somatic genetic variation that is known to exist at the cell and tissue level [32]. The construction of pan-cellular genome graphs is another direction to explore, especially as single cell multiomic technologies mature [33].

## Conclusions

Analyzing epigenomic datasets with personalized and graph genomes allows the recovery of novel ChIP-seq peaks many of which fall within genic regions and could differ between individuals. Although we focused this study on ChIP-seq, it is likely that these results will extend to other epigenomic assays such as ATAC-seq and whole-genome bisulfite sequencing. As we move toward profiling the epigenome of large human cohorts to study various phenotypes, it is likely that using personalized and graph genomes will reveal important loci that would have been missed otherwise.

## Methods

### Data

We selected NA12878 as a benchmark dataset due to the availability of phased variation calls from high coverage whole-genome sequencing (200×) [23] in addition to several de novo assemblies. FASTQs for H3K27ac, H3K4me1 marks, and a control (input) were downloaded from the ENCODE project [34]. The accession numbers for these samples are ENCFF000ASM, ENCFF000ASU, and ENCFF002ECP, respectively. We also used an ENCODE H3K4me3 dataset with accession number ENCSR057BWO for the IDR replicate analysis. To generate additional supporting results for ChIP-seq, we used a low pass NA12878 WGS dataset from IGSR [35] (SRR622461) [36]. Samples from the Blueprint project [37] were also selected due to the availability of phased variation calls from low pass whole-genome sequencing (8×) together with ChIP-seq datasets for the H3K4me1 and H3K27ac histone marks. In total, 151 H3K4me1 samples and 111 H3K27ac samples were used in this analysis.

### Preparing personalized genomes

Three different approaches were used to generate personalized genomes. First, vcf2diploid [7] was used to substitute the alternative sequence of the phased variation calls into the hg19 reference to create a MPG. The output are two FASTA files for each contig, forming the conventionally named maternal and paternal haplotypes. The contig FASTAs were concatenated according to their haplotype, resulting in one maternal and one paternal FASTA. It is to be noted that vcf2diploid does not process unordered contigs. Therefore, unordered contigs were removed from hg19 to ensure the same set of contigs between the standard and substituted versions. Also, vcf2diploid generates two chain files that allow the lifting of annotation tracks with coordinates in hg19 to the corresponding personalized haplotype using liftOver [38]. This is necessary since the incorporated indels shift the coordinates of the maternal/paternal haplotype relative to hg19.

The second approach, applied only to the NA12878 dataset, consisted of using de novo assembled genomes from the Pendleton [39] and two 10X Genomics assemblies [40] to create two DPGs. The 10X Genomics assembly includes two pseudo-haps named Hap1 and Hap2 that will be used as a de novo assembled diploid genome. In the case of the de novo assemblies, the chain files had to be produced from a BLAT [41] alignment between the de novo assembly and hg19 with the UCSC tool set [42]. This allowed the lifting of annotation tracks from the de novo assembly to the hg19 reference. The performance of DPG and MPG chain files was compared through the proportion peaks that failed to lift. Note that hg19 contains alternative contigs that represent some loci multiple times. In de novo assemblies, we expect loci to be represented only once. Therefore, the above analysis was performed on a hg19 version that was stripped of alternative contigs.

To allow alignment to personalized MPG or DPG FASTAs using bwa mem [43], an index was created using bwa index. A FASTA index was also created using samtools faidx [44] to compute the new chromosome sizes.

The third approach involved creating a reference graph genome by converting the linear hg19 reference to a graph format. A copy of this graph was then augmented with NA12878 variant calls, which yields the GPG. This was done with vg construct [18]. xg and GCSA2 graph indices were created with vg index to allow mapping reads with vg map.

### Aligning, peak calling, and annotating

To remove any effect of read length, all reads were trimmed to 36 bp using trimmomatic [45]. The trimmed reads were aligned using bwa mem to hg19 and each personalized haplotype FASTAs. After marking duplicates with picard [46], peak calling was done on the corresponding BAM files using MACS2 [47] with --nomodel and the --gsize parameter set to 80% of the assembly length. In graph genomes, peaks were called with Graph peak caller [19], a graph MACS2 implementation, by using the same linear genome size and the same fragment length parameter that was estimated by MACS2. The *q* value threshold (false discovery rate) was set at 0.05.

For each alignment, a coverage annotation was produced with bedtools bamtobed [48]. The output was a BED file listing all the aligned reads and their coordinates. Graph alignments (GAM) were surjected to BAM using vg surject and underwent the same procedure.

### Lifting annotations

In the case of DPGs, coverage and peak annotations were lifted from the DPG to hg19 using the tool liftOver. In the case of MPGs, the annotations were lifted from hg19 to the MPG. Therefore, this required the lifting of variant call annotations to the MPG in addition to the peak call and coverage annotations.

The variant call annotation was first converted from the VCF format to BED, separated by phase and type (SNP vs indel), and then lifted to the personalized haplotype. The outcome is a set of BED files listing the SNPs and indels separately for each respective haplotype.

liftOver was called with default arguments in BP samples, which require 95% sequence identity between lifted regions and target regions. This stringent -minMatch was not an issue since MPGs are almost identical to hg19 and virtually all peaks lift. In 10X and Pendleton samples, -minMatch was set 0.85 to reduce the number of unlifted peaks and reduce the number of false ref-only peaks. To evaluate lifting efficacy, the number of peaks that failed to lift was compiled for every sample. Once tracks are lifted to a common coordinate system, it becomes possible to overlap and compare the annotations from the personalized haplotype and the hg19 standard reference using bedtools.

Graph annotations are readily surjected onto hg19 using built-in functionality in vg and Graph peak caller.

### Overlapping annotations

The lifted or surjected peak call annotations were overlapped using bedtools intersect and bedtools subtract. Peaks resulting from the intersect of the personalized and the hg19 peak tracks were categorized as *common*. Peaks resulting from subtracting the hg19 track from the personalized track were categorized as *personal-only*. Similarly, peaks resulting from subtracting the personalized track from the hg19 track were categorized as *ref-only*. If a peak in the personalized genome shows any partial overlap with a peak in the reference genome, it is labeled as *common*. The end result is a set of three BED files for each personalized genome containing the common peaks, the personal-only peaks, and the ref-only peaks. Note that these definition depend solely on the peak call annotation and do not take into consideration the read depth of those peaks.

The number of variation calls in each peak was calculated. The corresponding indel and SNP tracks were intersected with the track of each category of peaks using bedtools intersect -c to list the number of variations overlapping each common, personal-only, and ref-only peak.

Furthermore, the peak tracks were overlapped with the coverage tracks of the personalized and hg19 versions of the alignment using bedtools intersect -c. The output is the original peak track with an additional field listing the number of reads in each peak. As a result, the number of reads in regions corresponding to the peaks is known in the reference alignment and the personalized alignment.

### Finding peaks with skewed coverage

To find peak called regions that have significant differences between their hg19 and personalized coverages, a statistical test was needed. This comparison is similar to differential expression in that read counts are compared between two conditions: the hg19 reference and the personalized assembly. For the purpose of differential expression, technical variation that occurs during the preparation of different libraries is known to be underestimated by Poisson-based tests (overdispersion) [49]. However, unlike differential expression, our read counts are not compared between multiple sequencing experiments done under the two conditions. Instead, there is only one dataset that was aligned to two different assemblies, which implies that biological and technical variation is not present here in the same way. Therefore, we simply used a *χ*^2^ test with a significance value *α* of 0.05 to detect peaks with skewed coverage. We obtained an identical result with the edgeR package [50] by setting the dispersion parameter to 1×10^−3^ (near 0). Peaks with null coverage in one of the alignment versions were artificially assigned one read to allow applying the test. Peaks with insignificant differences were placed in the no-skew category. An overview of the above steps can be found in Figures S15a and S15b.

Peaks that had significant differences with a higher coverage in hg19 than in the personalized haplotype were categorized as ref-skewed. Similarly, peaks that had a higher coverage in the personalized genome than in the reference were categorized as personal-skewed.

### Characterizing altered peak calls

To quantify the fraction of AP calls, the number of ref-only and personal-only peaks was counted and then divided by the total number of peaks to obtain their frequency relative to the total number of peaks in their sample. For each sample, the set of all peaks was divided into mutually exclusive categories according to the combination of overlapping variation calls (SNPs only, indels only, SNPs and indels, none). The same was repeated for ref-only and personal-only peaks. For any given variation category, the counts of ref-only and personal-only peaks were divided by the sample wide peak count of the given category to obtain the probability that the peak call could be affected by that specific combination of variations. At the same time, the mean peak widths were recorded.

For DPGs, we counted the number of hg19-relative variant calls overlapping common, ref-only, and personal-only peaks. We did this to check whether ref-only peaks and personal-only peaks remained enriched in hg19-relative variation calls compared to common peaks, despite the fact that they originate from peak calls in a de novo assembly and not hg19 itself.

We also counted the overlaps of altered peaks in DPGs with SDs and repeats from the RepeatMasker annotation. Repeats were first grouped by family. Confident peaks were selected by removing any peak with a log(MACS2 score) < 4.0. This value was chosen because it excludes uncertain and uninteresting peak calls and most APs generated by MPGs.

Logistic regression was performed on NA12878 H3K4me1 peaks with AP/common as a binary response variable and peak width, SNP count, and indel count as covariates using the glmnet [51] R package. Ref-only and personal-only peaks were coded as AP=1, and common peaks were coded as AP=0. Lastly, common peaks were downsampled to the number of AP calls to avoid unbalanced classes. Since the fitting algorithm is non-deterministic, we ran cv.glmnet 1000 times and reported the median coefficient values.

In the H3K4me3 replicate analysis, Graph Peak Caller was run on the alignment of each replicate and also on the merged alignments. IDR was run on the peaks of each replicate to obtain corrected peaks to be compared against the peaks of the merged alignments.

The histone mark correlations were generated with HOMER [52] to support altered peaks with orthogonal data.

### Comparing WGS alignments between genomes

If peak track differences occur between two assemblies, they should be corroborated by differences in the mapping of a sufficient number of reads between their raw alignments. That is, the proportion of reads with different mappings between the reference and the personalized genome should be considerable. To show this, we used Jvarkit cmpbamsandbuild [53] to compare the DPG and MPG alignments of the low pass NA12878 whole-genome dataset to hg19. The same comparison was done between the reference and the paternal NA12878 MPG. To compare the GPG and the reference graph alignments, vg gamcompare was used instead. For unequal mappings, we considered reads that are mapped more than 100 bp apart, reads that are mapped in one build but not the other, and reads that fail to lift between assemblies. We add these proportions to obtain the final proportion of changed mappings. The IGSR WGS dataset was chosen instead of a ChIP-seq dataset because we expect a more uniform coverage of genomic regions.

### Finding replicated peaks among MPGs, DPGs, and GPGs

To get the replicated calls between the DPG and the MPG approaches, the personalized tracks needed to be lifted to a common coordinate system in hg19. This is necessary because the MPG APs were computed in MPG coordinates, while the DPG and GPG APs were computed in hg19 coordinates. To do so, chain files were created through the previous BLAT method to lift the MPGs to hg19. Once the tracks of ref-only and personal-only peaks respective to the MPGs were lifted to hg19, GenomicRanges [54] was used to calculate the pairwise overlap of peak calls between the three approaches and identify peaks that are replicated with at least two of the three methods. This package was also used to characterize the position of peaks relative to genes in the UCSC gene annotation. A Venn diagram was produced for personal-only calls, ref-only calls, and all peak calls using nVenn [55].

## Supplementary information

**Additional file 1** Supplements, contains supplementary figures and tables.

**Additional file 2** Review history.
