# Supplementary material for: Personalized and graph genomes reveal missing signal in epigenomic data
Source: Genome Biol. 2020 May 25;21:124. doi: 10.1186/s13059-020-02038-8 (PMC7249353; doi:10.1186/s13059-020-02038-8)
Supplement: Supplementary file 1 — Additional file 1 Supplements, contains supplementary figures and tables. [file 13059_2020_2038_MOESM1_ESM.pdf]

Supporting figures

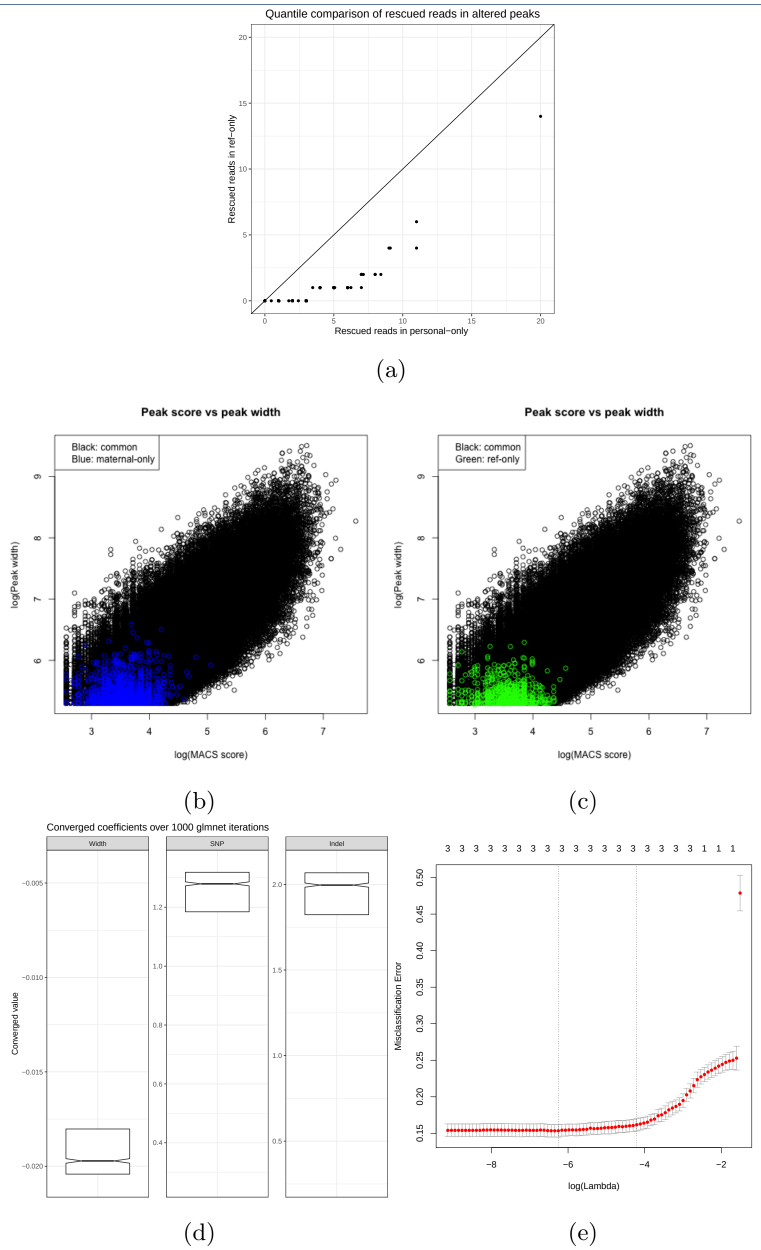

Figure S1: a) Quantile-quantile comparison between the distribution of rescued reads in ref-only and personal-only distributions. b) Confidence and width of the NA12878 maternal MPG altered peak calls (H3K4me1). Peaks called only in the maternal MPG against the common peak background. c) Peaks called only in hg19 against the common peak background. d) Distributions of converged coefficients for the width, SNP count and indel count terms in the `glmnet` logistic regression model. Median coefficients are 0.19 for width, 1.29 for SNP 1.9 for indels. e) The chosen regularization parameter corresponds to the minimum misclassificaton error during k-fold cross-validation. This model achieves a 0.15 misclassificaton error.

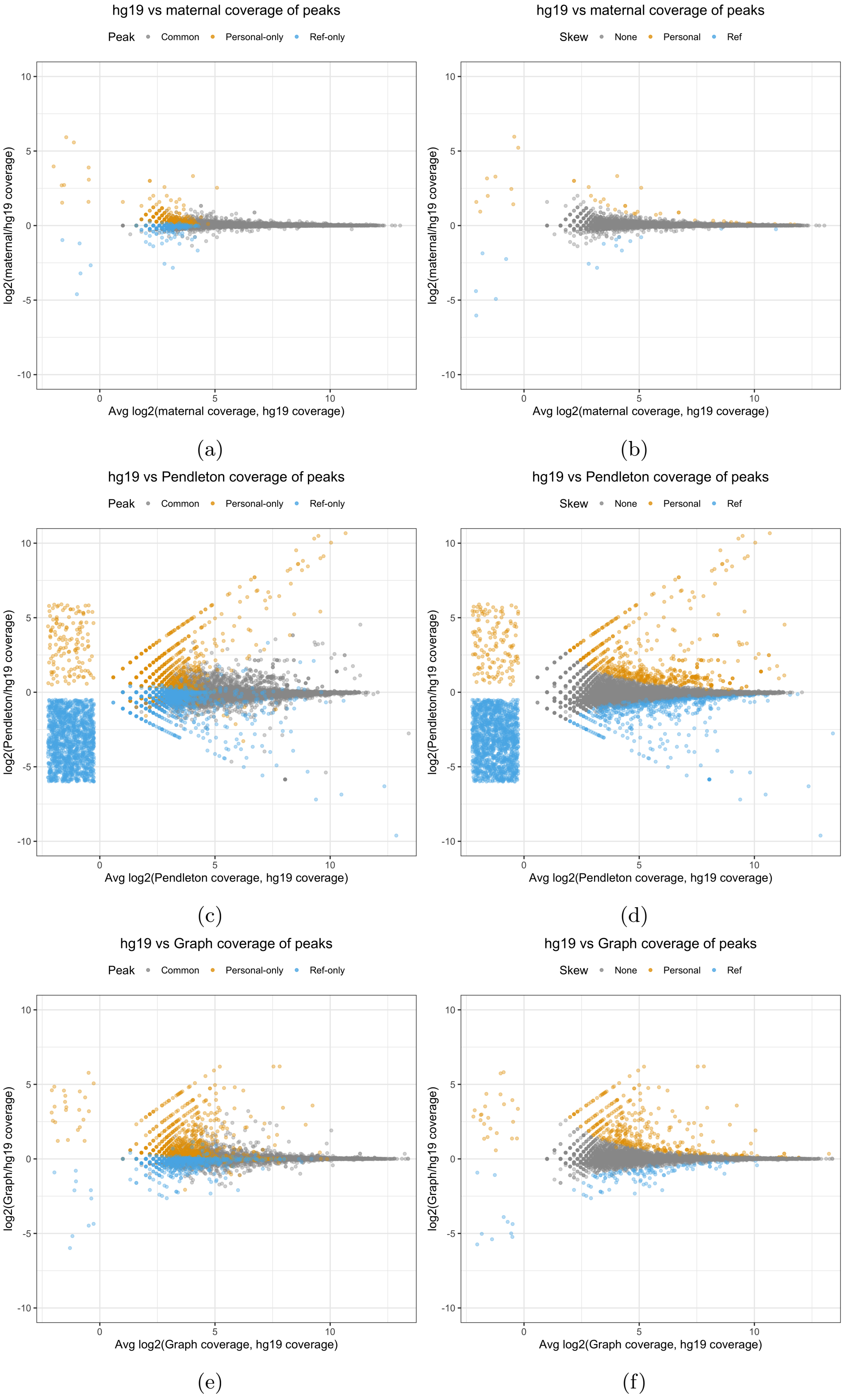

Figure S2: MA plots for H3K27ac analogous to H3K4me1.

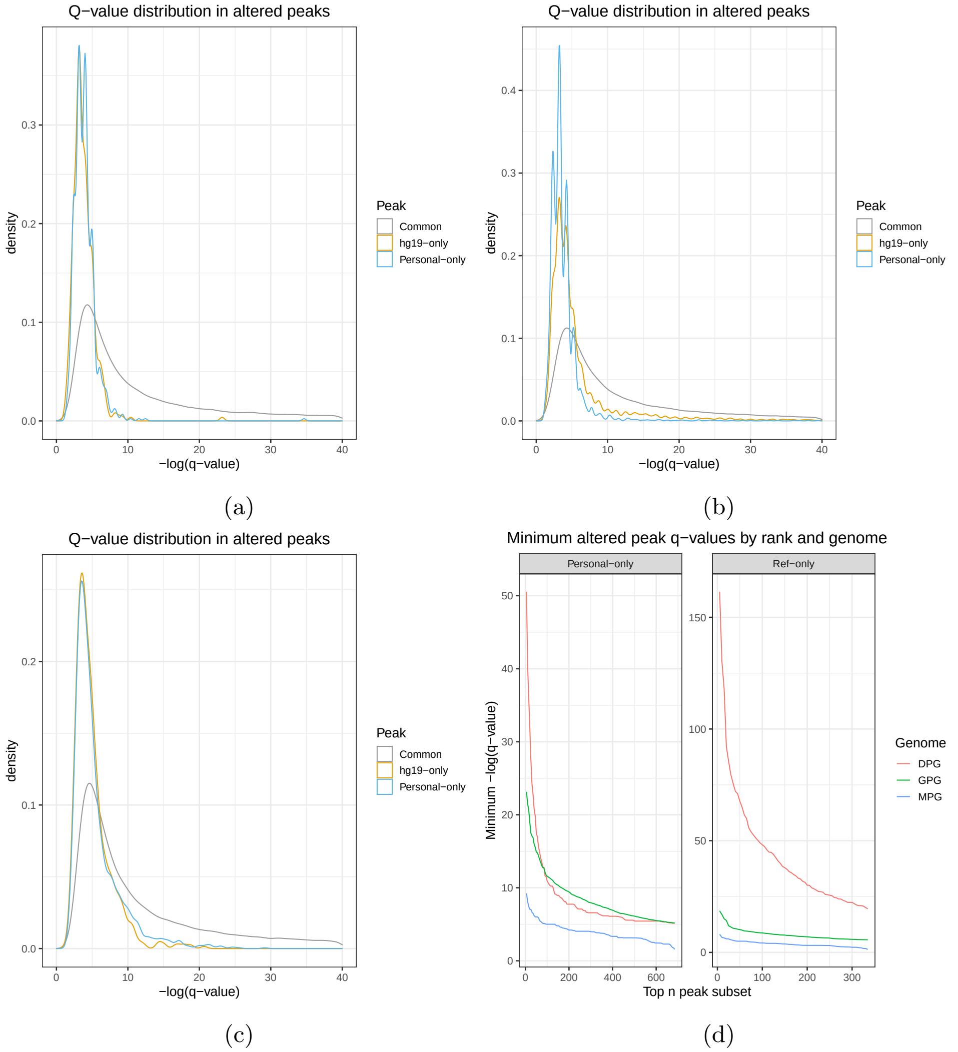

Figure S3: Q-value distributions for H3K27ac analogous to H3K4me1. a) In MPGs. b) In DPGs. c) In GPGs. d) Comparison by rank.

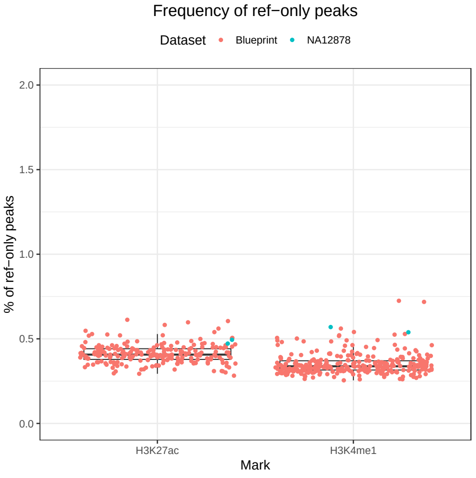

(a)

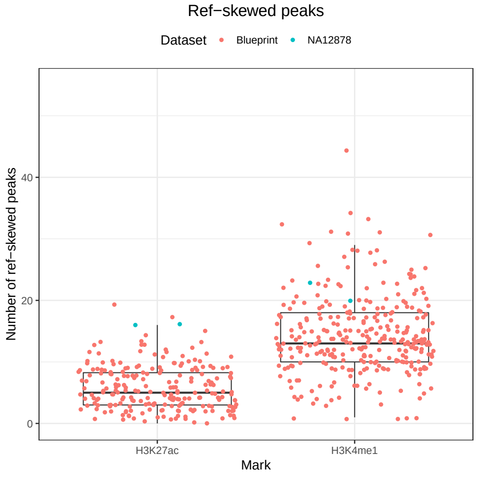

(b)

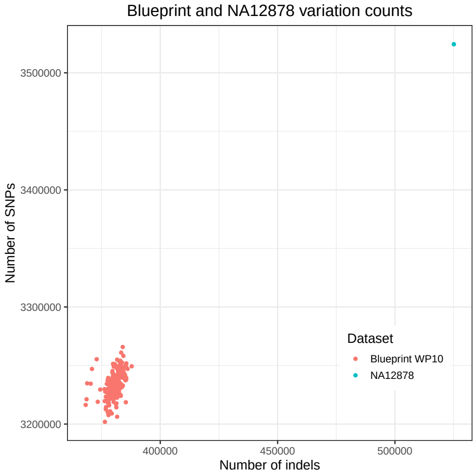

(c)

Figure S4: Comparison between NA12878 and Blueprint samples. a) Proportion of peaks that are called only in the reference. b) Number of peaks with higher coverage in the reference than the MPG. c) Difference in number of variation calls (SNPs and indels) between Blueprint samples and NA12878.

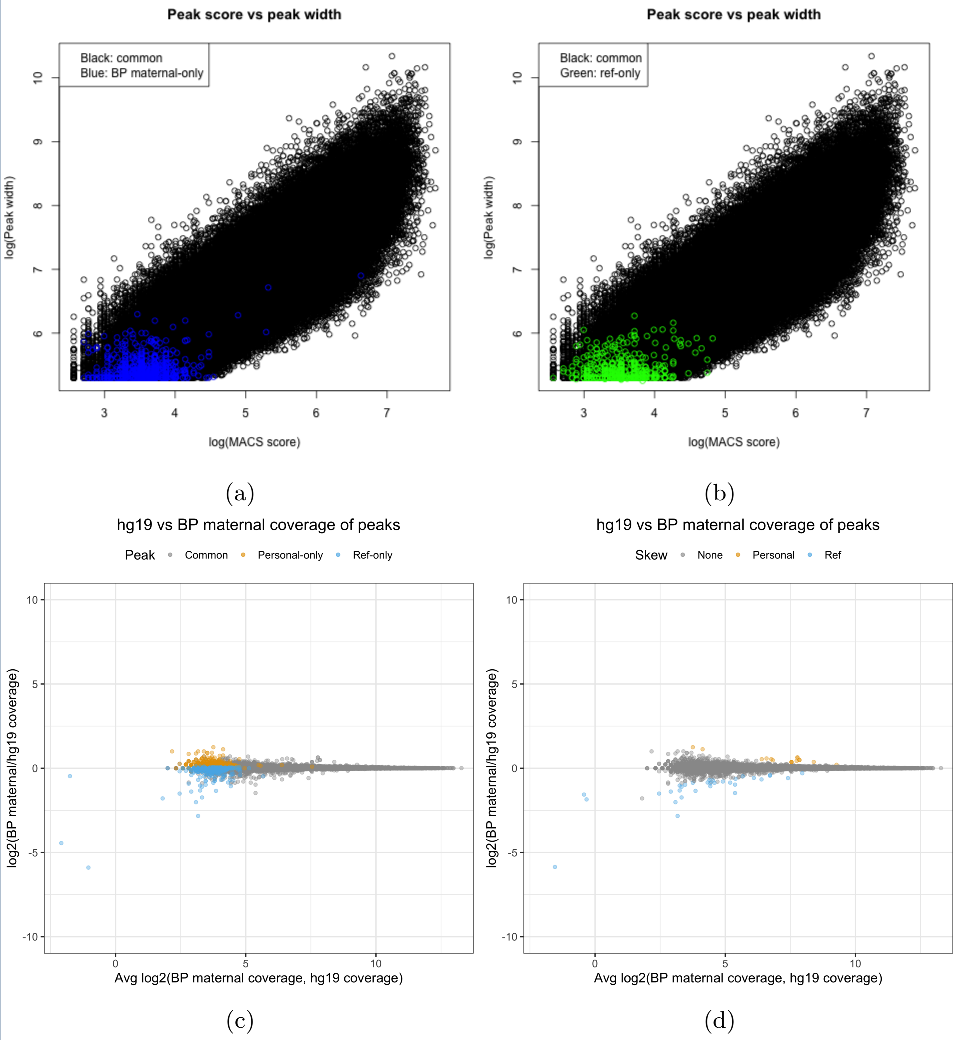

Figure S5: Characterization of AP calls in the maternal MPG of a typical Blueprint sample (H3K4me1).

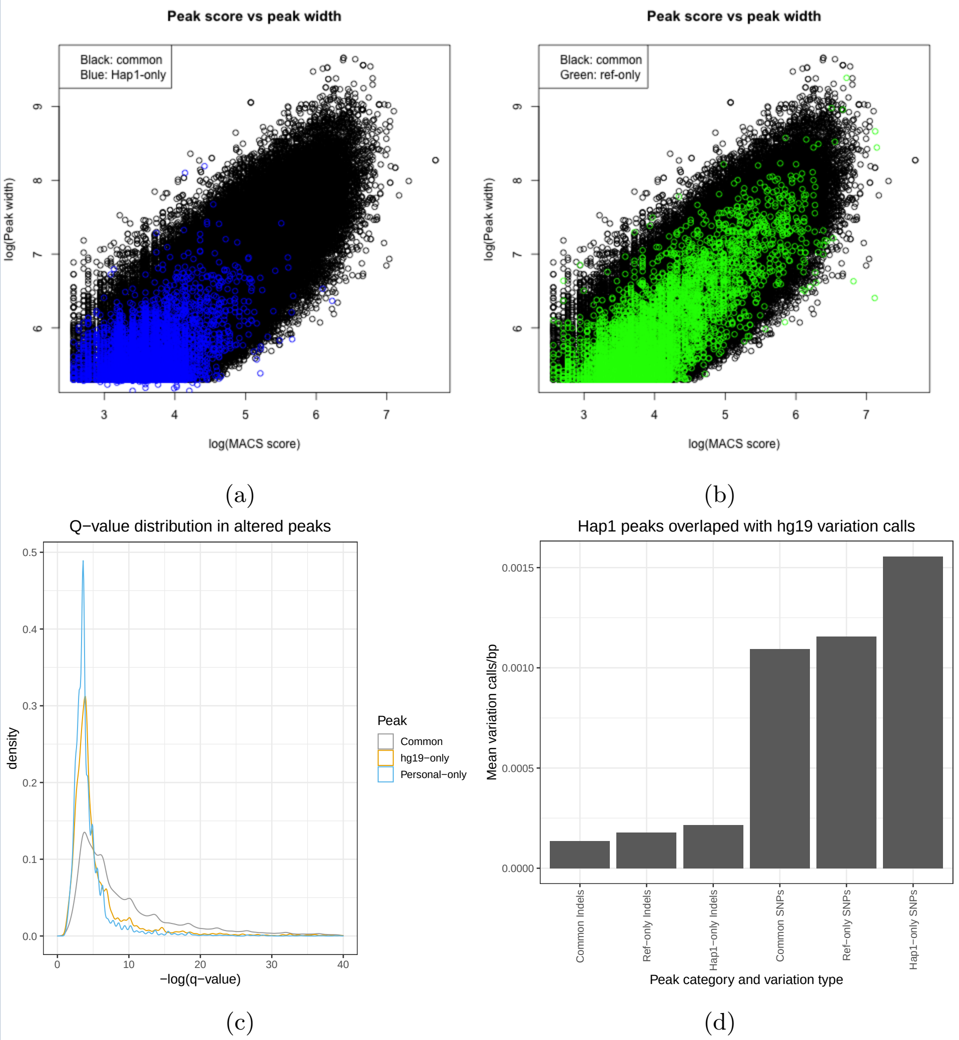

Figure S6: Confidence and width of the Hap1 DPG altered peak calls (H3K4me1). a) Peaks called only in Hap1 against the common peak background. b) Peaks called only in hg19 against the common peak background. c) Q-value distributions of the same peaks. d) Hap1-only peaks are enriched in hg19-relative variant calls relative to common peaks.

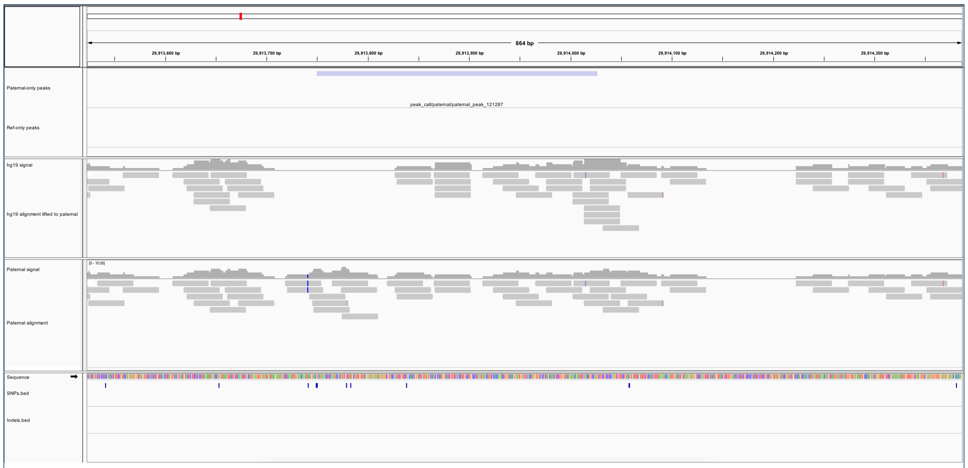

(a)

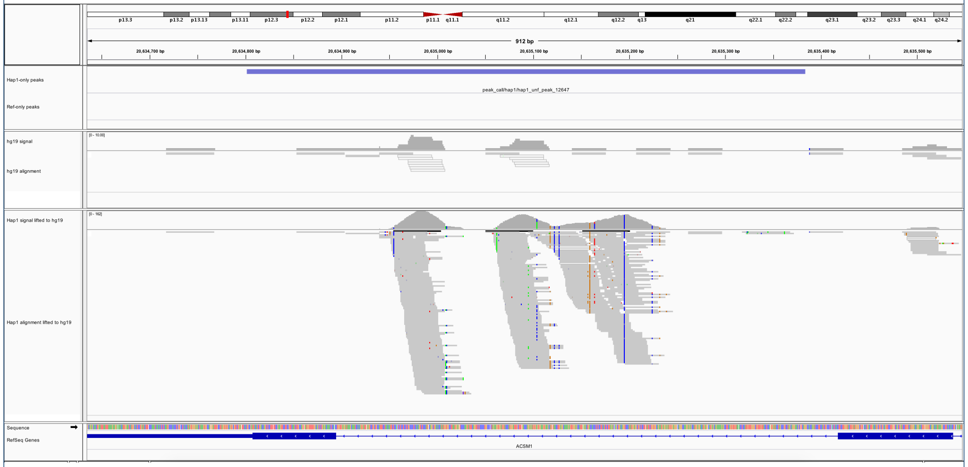

(b)

Figure S7: a) Typical personal-only peak in a NA12878 MPG. The small variations have a visible effect on only a few reads. (b) SD-free personal-only peak in the Hap1 DPG. Large scale changes in coverage become apparent with this approach.

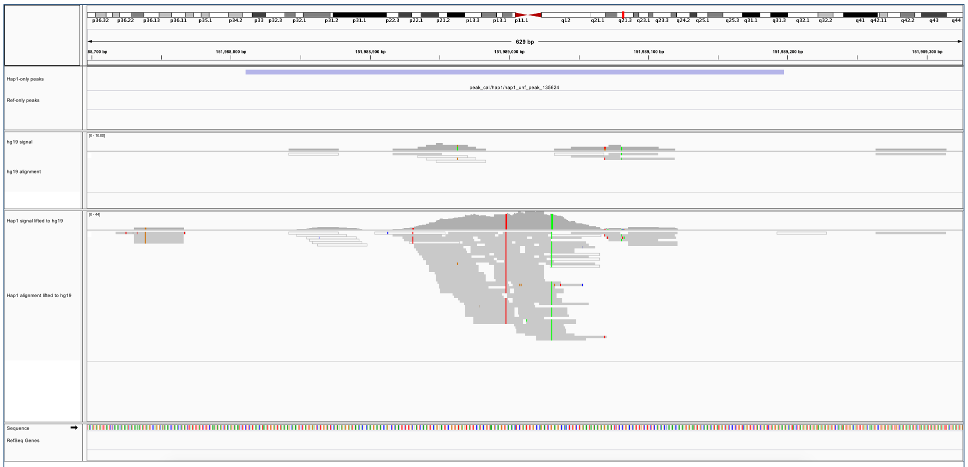

Figure S8: Hap1-only peak free of repeats and segmental duplications. Viewing this region in the UCSC genome browser shows overlaps with the hg19 self chain.

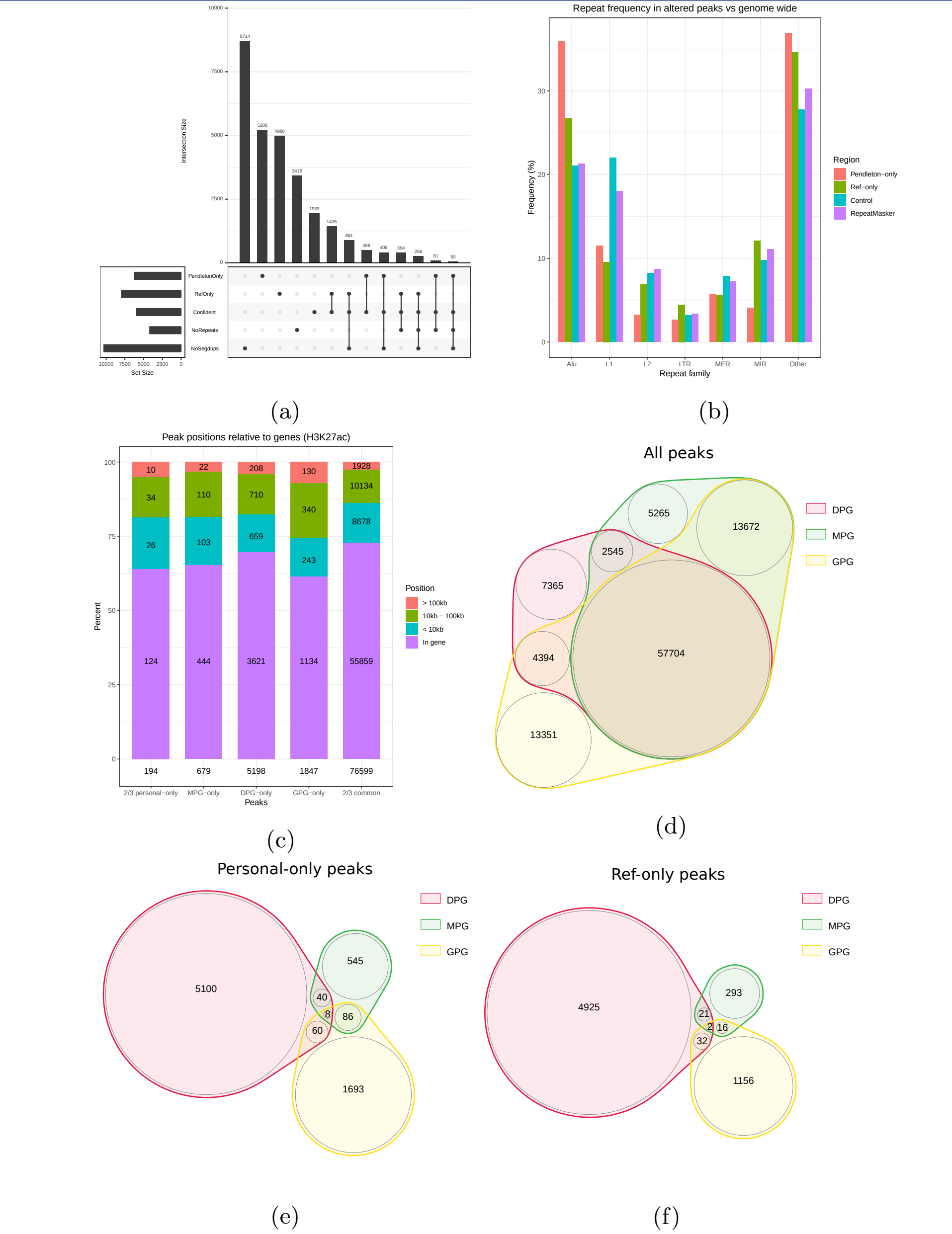

Figure S9: Plots for the H3K27ac mark that are analogous to H3K4me1. a) Summary of the overlap between altered peaks, confident peaks, repeats and segmental duplications. b) Frequency of repeat families within altered peaks compared to genome wide. The control is random intervals with the same width distribution as altered peaks. c) Distribution of gene relative positions of personal-only peaks among all genomes. The DPG is the Pendleton assembly. d) Overlap of all peak calls. e) Overlap of altered personal-only peak calls. f) Overlap of ref-only peak calls.

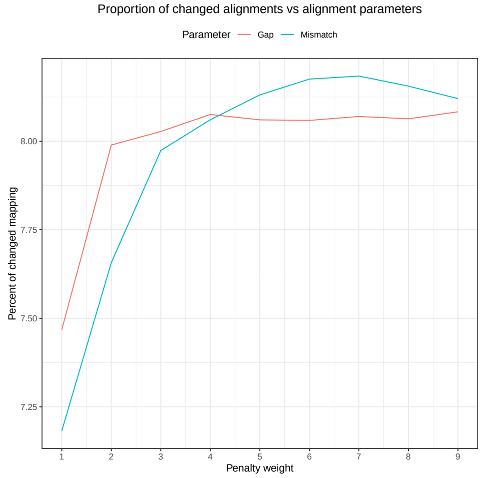

(a)

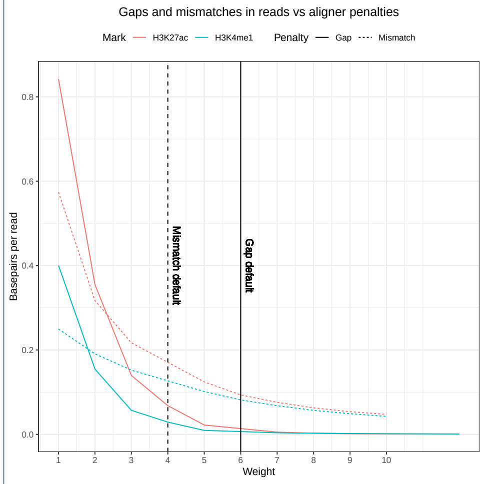

(b)

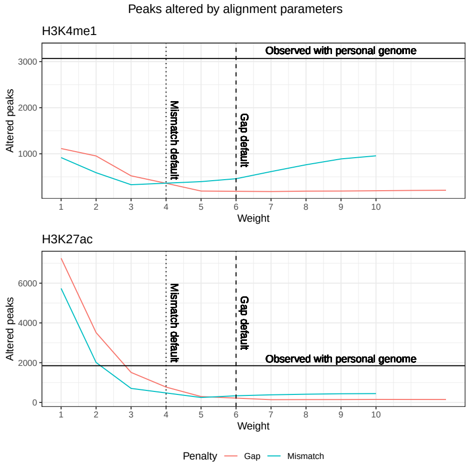

(c)

Figure S10: a) Impact of the gap and mismatch alignment penalties on the proportion of changed mapping between the reference and the NA12878 GPG. This proportion only falls by 1% even when using the most lenient alignment penalties. b) Number of mismatch and gap events per read with respect to alignment mismatch and gap penalties. Relaxing penalties when aligning to the reference admits many poor quality alignments. c) Number of altered peaks created by aligning reads to the reference genome and varying gap and mismatch penalties in H3K4me1 (top) and H3K27ac (bottom). The lowest penalties can generate more peaks than a personalized genome by admitting many poor quality alignments that would be rejected with default parameters (especially in the H3K27ac dataset). Personal genomes generate thousands of APs without admitting poor quality alignments.

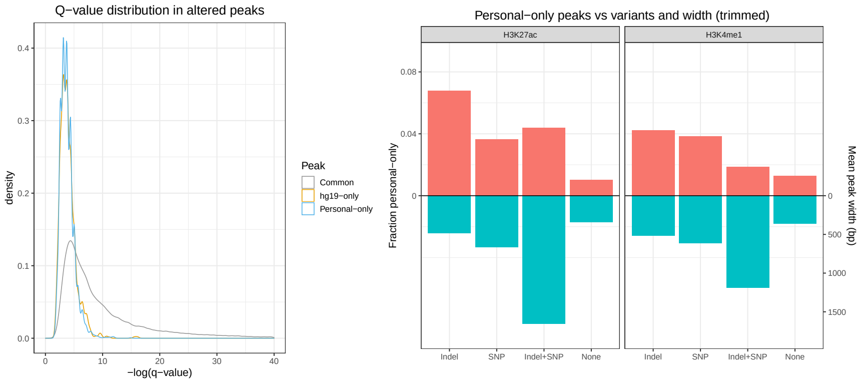

(a) (b)

Figure S11: a) Q-value distribution of H3K4me1 altered peaks in GPGs. b) Replicated estimates of the probability that variants will cause an altered peak in GPGs.

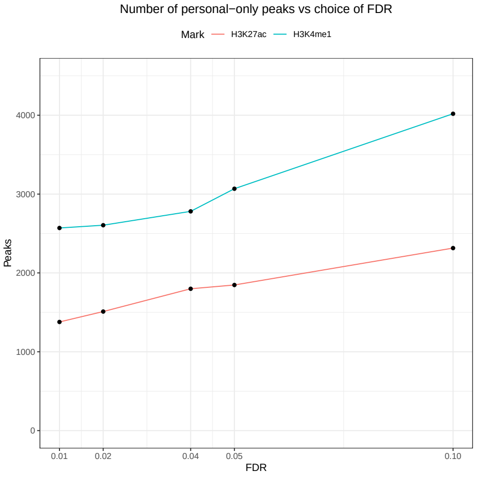

Figure S12: The number of APs calls increases when relaxing the false discovery rate (FDR) parameter from 0.05 to 0.10. Conversely, the number of APs decreases by roughly a quarter as the FDR approaches 0.01. This decrease is mild given that the FDR parameter is five times more stringent.

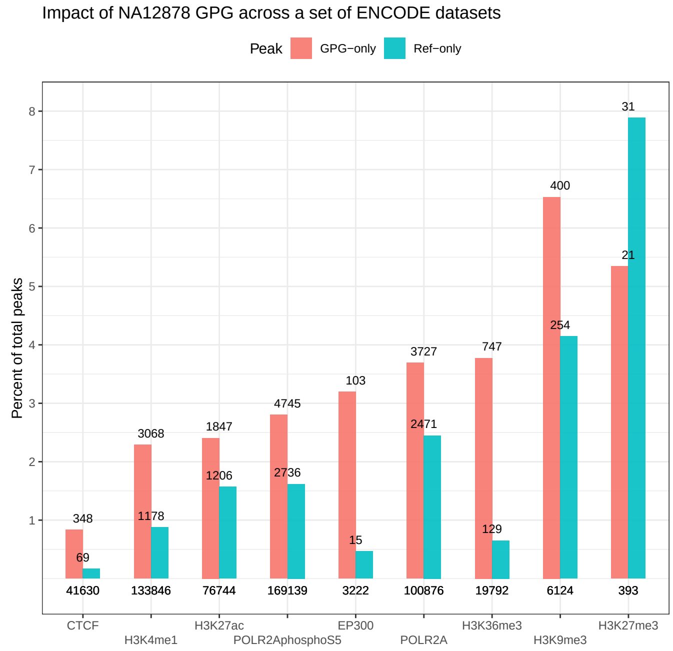

Figure S13: Proportion of personal-only and ref-only peaks generated by different NA12878 ENCODE dataset. Top and bottom annotations show the numbers of APs and total peaks respectively. Histone marks, POLR2A and EP300 show similar or higher proportions of altered peaks while the transcription factor CTCF shows the lowest impact. H3K27me3 shows an unusually small number of total peaks, which may explain a larger than usual proportion of APs, especially for ref-only peaks.

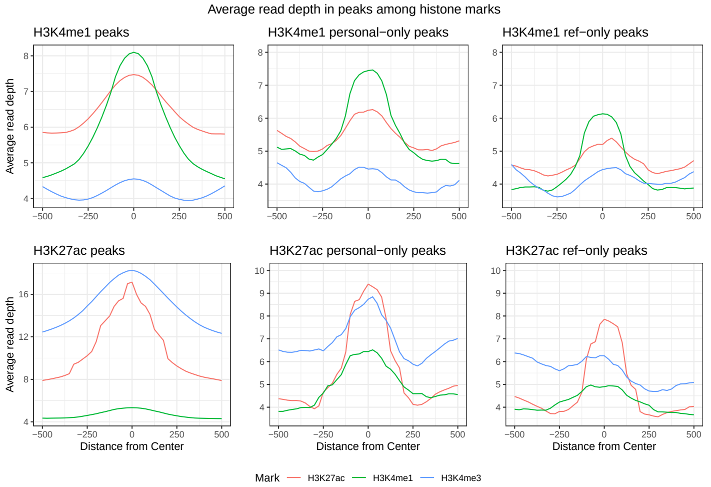

Figure S14: Average H3K4me1, H3K27ac and H3K4me3 read depth of common, personal-only and ref-only peak calls in the NA12878 GPG. Histone peaks show correlation patterns characteristic to the histone code. Personal-only calls show an increased read depth compared to ref-only calls.

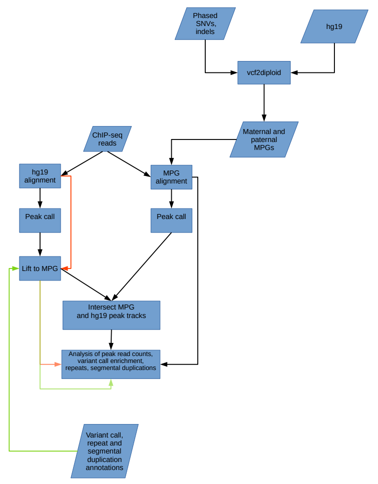

(a)

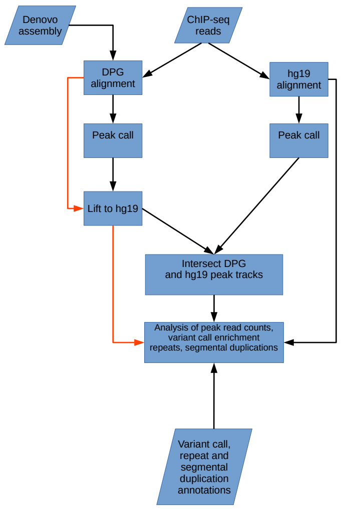

(b)

Figure S15: Flow chart of analysis for a) modified personal genomes and b) denovo personal genomes.

Supporting tables

Case (NA12878 MPG)	36bp reads		Different
Failed liftover	1947562	1.1%	Yes
Lifted coordinates	167201030	90.4%	-
↪ Equal in hg19, MPG	162854003	88.0%	No
↪ Unequal in hg19, MPG	4347027	2.4%	Yes
Unmapped in hg19, mapped in MPG	122163	0.07%	Yes
Mapped in hg19, unmapped in MPG	46565	0.03%	Yes
Unmapped in hg19, unmapped in MPG	15601588	8.4%	No
Total	184918908	100%	3.6%

(a)

Case (NA12878 DPG)	36bp reads		Different
Failed liftover	8968154	4.8%	Yes
Lifted coordinates	160169017	86.6%	-
↪ Equal in hg19, Hap1	152748687	82.6%	No
↪ Unequal in hg19, Hap1	7420330	4.0%	Yes
Unmapped in hg19, mapped in Hap1	156895	0.08%	Yes
Mapped in hg19, unmapped in Hap1	1683155	0.91%	Yes
Unmapped in hg19, unmapped in Hap1	13941687	7.54%	No
Total	184918908	100%	9.79%

(b)

Case (NA12878 GPG)	36bp reads	
Unequal mapping	15378825	8.3%
Equal mapping	169540083	91.7%
Total	184918908	100

(c)

Table S1: a) Breakdown of WGS 36bp read alignment comparison between the reference and the NA12878 paternal MPG. The proportion of failed liftover, mapped/unmapped differences and unequal coordinate reads is 3.6%. This result is near identical in the both NA12878 MPGs. b) In the same comparison between the Hap1 DPG and the reference, the proportion is 9.79%. c) Comparing the alignments to the graph reference and the augmented NA12878 graph yields a proportion of 8.3% of reads with unequal mapping.

Genome (NA12878)	Personal-skewed	Ref-skewed	Mark
MPG, Maternal	30	23	H3K4me1
MPG, Paternal	32	20	H3K4me1
DPG, Hap1	2216	3152	H3K4me1
DPG, Hap2	2221	3162	H3K4me1
DPG, Pendleton	1518	4320	H3K4me1
GPG	279	244	H3K4me1
MPG, Maternal	42	16	H3K27ac
MPG, Paternal	50	16	H3K27ac
DPG, Hap1	1497	1416	H3K27ac
DPG, Hap2	1456	1422	H3K27ac
DPG, Pendleton	781	1882	H3K27ac
GPG	411	127	H3K27ac

Table S2: Number of peaks with skewed coverage in NA12878 MPGs and DPGs for H3K4me1 and H3K27ac marks. The read length across all rows was kept at 36bp.

Version	Mark	common	personal-only	ref-only
MPG, Paternal	H3K4me1	129496	746 (0.6%)	456 (0.4%)
MPG, Maternal	H3K4me1	129497	747 (0.6%)	455 (0.4%)
MPG, Paternal	H3K27ac	46830	336 (0.7%)	196 (0.4%)
MPG, Maternal	H3K27ac	46831	335 (0.7%)	195 (0.4%)

Table S3: Average number of altered peak calls in MPGs for the Blueprint samples for the same marks.

Case (NA12878 MPG)	100b reads		Different
Failed liftover	2030979	1.1%	Yes
Lifted coordinates	175123570	94.7%	-
↪ Equal in hg19, MPG	170876933	92.4%	No
↪ Unequal in hg19, MPG	4246637	2.3%	Yes
Unmapped in hg19, mapped in MPG	39169	0.02%	Yes
Mapped in hg19, unmapped in MPG	19307	0.01%	Yes
Unmapped in hg19, unmapped in MPG	9632679	5.2%	No
Total	184918908	100%	3.43%

(a)

Case (NA12878 DPG)	100bp reads		Different
Failed liftover	9769845	5.3%	Yes
Lifted coordinates	165311184	89.4%	-
↪ Equal in hg19, Hap1	158400964	85.7%	No
↪ Unequal in hg19, Hap1	6910220	3.7%	Yes
Unmapped in hg19, mapped in Hap1	49997	0.03%	Yes
Mapped in hg19, unmapped in Hap1	749507	0.4%	Yes
Unmapped in hg19, unmapped in Hap1	9038375	4.9%	No
Total	184918908	100%	9.43%

(b)

Case (NA12878 GPG)	100bp reads	
Unequal mapping	14902323	8.06%
Equal mapping	170016585	91.94%
Total	184918908	100

(c)

Table S4: a) Breakdown of WGS 100bp read alignment comparison between the reference and the NA12878 paternal MPG. The proportion of failed liftover, mapped/unmapped differences and unequal coordinate reads is 3.43%. This result is near identical in both NA12878 MPGs. b) In the same experiment with the Hap1 DPG and the reference, the proportion is 9.43%. c) Comparing the alignments to the graph reference and the augmented NA12878 graph yields a proportion of 8.06% of reads with unequal mapping.

Ref-only peaks	Number	Contain segmental duplications	Percent
Null coverage in Hap1	2547	1781	69.9
↪ Confident	1490	1062	71.3
Positive coverage in Hap1	4208	267	6.3
↪ Confident	574	98	17.1

Table S5: Ref-only peaks with null coverage in the Hap1 DPG are extremely enriched in segmental duplications.

Peak type	MACS2 (FDR 0.05)	IDR
Reference peaks	29484	21764
NA12878 GPG peaks	29491	21785
Personal-only peaks	293 (0.99%)	138 (0.6%)
Reference-only peaks	77 (0.26%)	85 (0.39%)

Table S6: Summary of IDR results for the NA12878 ENCODE H3K4me3 dataset with replicates.
