# Supplementary material for: Personalized and graph genomes reveal missing signal in epigenomic data
Source: Genome Biol. 2020 May 25;21:124. doi: 10.1186/s13059-020-02038-8 (PMC7249353; doi:10.1186/s13059-020-02038-8)
Supplement: Supplementary file 2 — Additional file 2 Review history. [file 13059_2020_2038_MOESM2_ESM.docx]

Review History

**First round of review**

**Reviewer 1**

**Are you able to assess all statistics in the manuscript, including the appropriateness of statistical tests used?**

Yes, and I have assessed the statistics in my report.

**Comments to author:**

Reviewer 1

Referee report for “Personalized and graph genomes reveal missing signal in epigenomic data”

This manuscript evaluates the impact of choice of reference genome for alignment in ChIP-seq analysis. Currently, the standard practice relies on reference human genome for identifying peaks, and modified personal genomes for identifying allele specific occupancy. However, there has not been systematic efforts in investigating the pitfalls of using reference genomes. The paper considers three alternatives to reference genome: modified personal genomes that are obtained by modifying the reference genome by replacing the SNPs, denovo assembled genome, and finally graph personalized genome which provides an alternative between the first two choices. While I appreciate the effort, there are a number of significant weaknesses pertaining the analyses.

1)	The overall conclusion seems to be that using a personalized genome identifies a small number of additional peaks and removes an even smaller number of peaks that were identified by the reference genome. While this is a reasonable observation, given the instability of peak callers, these percentages may not be significant (or they may be in the order the variation one tends to see if the alignment or peak calling parameters are slightly varied).

The manuscript uses MACS for peak calling, but does not specify what level of false discovery rate is used. One issue with MACS and other peak callers is that they cannot reliably control FDR. The ENCODE project developed IDR as an alternative. Carrying out these investigations with IDR would be more robust and conclusive.

2)	Another concern is that it is not clear whether the APs (altered peaks) can be recovered by varying the thresholds of peak calling. In other words, perhaps the peaks specific to personalized genomes are just below the peak-calling threshold when the data is analyzed with the reference genome (e.g., they are not identified at 5% FDR but make it to the list in 10%). In this case, APs arise due to change in ranking of the peaks and one would need to argue that the new ranking is somehow more meaningful. Authors should make a clear distinction between APs that will never be recoverable (not even with higher depth) unless one uses personalized genomes. Fig4a depicts such peaks as a smear in the MA plot, but it is not clear whether these are as reproducible as the ones with some reads with the reference analysis.

3)	A related issue is also the impact of alignment on peak calling. It is important to show that the variation observed with the change of alignment genome is larger than that would arise by changing the alignment parameters.

4)	It is not clear how accurate the alignments are with GPGs or MPGs compared to reference genomes? A computational experiment for quantifying this is warranted.

5)	It is also important to investigate whether APs specific to personalized genomes are more supported by external data (ATAC-seq, RNA-seq or some other) compared to lost APs (reference genome specific peaks).

6)	What is the impact of personalized genomes on allele specific quantification? Are the authors seeing that some of the APs specific to personalized genomes would not have been detected by allele-specific quantification.

**Reviewer 2**

**Are you able to assess all statistics in the manuscript, including the appropriateness of statistical tests used?**

No, I do not feel adequately qualified to assess the statistics.

**Comments to author:**

Review on:

Personalized and graph genomes reveal missing signal in epigenomic data

By Groza et al.

* Summary

The authors investigate whether a graph representing a reference genome and known variations helps calling ChIP-seq peaks compared to when the analysis is performed against a single reference genome.

This study belongs to a group of studies that seek to delineate the improvements brought by using genome graphs. It is a natural question since the sequencing of multiple individuals brings key knowledge on individual variations. Beyond the single linear reference genome (which is the current gold standard for read analyses), three alternative personalized graphs are considered: modified personalized genome (MPGs), graph genomes (GPG), and de novo assembled personal genomes (DPGs). In this order, each graph incorporates more variability, and provides higher fidelity to the individual genome.

* Pedagogical aspects

The use of genome graphs is not common place and may puzzle a lot of readers.

A broader general introduction about this concept, and its variants would help the readers to grasp the potential impact of genome graphs, which will likely become standard within 5-10 years from now (when available of course).

Before entering the results section, please provide a clear definition (at least description) of each type of genome graphs, and explain their differences, which could be illustrated with well chosen examples. For instance, Figure 1 does not illustrate the fact that you use phased variants for creating the MPG. The legend should be more explicit regarding the strike G in the MPG box. Moreover, a pointer to a recent review:

Computational Pan-Genomics: Status, Promises and Challenges

Briefings in Bioinformatics, Vol 19(1), p. 118-135, doi: 10.1093/bib/bbw089 2018

would complete these explanations and allow the readers to extend their understanding of the topic.

* General major remarks.

1. The peak calling is evaluated with two distinct epigenetic marks.

Can you give a rationale behind this choice? Why do you consider only two? Could you justify that two is enough for this study, which seeks to yield general conclusions?

In any case putting some background knowledge about each mark in your introduction can help the reader grasping the context.

2. Species

Similarly, this works concentrates on Human data, which may be justified by the quality of reference and the availability of high quality known variations.

3. In my view, most of the results follows from the difference in read mapping, rather than in peak calling. It has already been shown that read mapping against a sequence graph rather than against a linear reference sequence, strongly impacts read mapping (see e.g. BMC Bioinformatics doi:10.1186/s12859-016-1103-9, 2016, and later works citing this one).

I would find valuable that you detail the read mapping results against the three graphs and evaluate the impact of read mapping parameters on these, before investigating the peak calling impact. It is necessary to understand the obtained results.

Your observation from p 5 l 17 and beyond "We found, as expected, that as the read length increases, APs become less likely (Fig 3d). We repeated the NA12878 WGS alignment comparison with the longer 100bp reads to gain some insight on why this happens (Table S4a). Compared to the shorter reads (Table S1a), the longer reads show a proportionally small decrease in aligned reads with unequal mappings." really supports the view that mapping is the key step in the whole analysis in terms of impact.

Other observations later p7 l11-12 suggest that testing mappers that can better handle indels could change the results. Could you consider such mappers (e.g. GMAP) in your tests?

4. Beyond mapping, the peak calling parameters may impact the results. Could you please investigate further the robustness of your results with respect to major peak calling parameters?

5. In your description, the DPG is the ultimate graph solution in terms of genome fidelity. Maybe this is not completely true. This view disregards the variations at organs or cell levels. What about the pan-cellular graphs built from multiple single cell whole genome samples?

Similarly, to extend MPGs, how could you incorporate the relative frequency of known variations in your graph to improve predictions?

6. Reproducibility

I have not attempted to reproduce your results. The description provided in the Methods section give a broad view of your computational experiments. However it is not sufficient for full reproducibility. Could you provide in supplementary material a Snakemake script (or several) that copes with software installation and can be run to fully reproduce your quantitative results and figures?

7. Do your findings have biological implications?

Sentences like p8 - l8. You support your claim with one example. In my view, it is insufficient.

I am persuaded that sequence graphs makes a difference compared to a single reference genome, but i have the impression that your results do not suggest this difference have a critical impact on biological considerations. This is a difficult question.

Looking at differences e.g. in enhancer regions that would impact transcription initiation rate, could help showing that ChIP-seq peak calling is improved thanks to genome graphs.

* Precise remarks

- p2 l18 (MPG) is singular

- p3 l54 "Ideally, peaks that have AP calls should also have a skewed coverage." Could you be more precise and explain why it'd be ideal?

- p3 results: clarify the notion of personal-only, ref-only and common peaks, as well as Altered peaks, given that peaks may inexactly overlap, may get similar, but still unequal coverage.

- p3 l59: 30 out of ??

- p4 l7 define "lower confidence"? Explain how you compare peaks' confidence

- p4 l52-53 "as the NA12878 variant call set (3.5M SNPs, 0.5M indels) is richer than Blueprint (approximately 3.25M SNPs and 375M indels per sample, Fig S4c)." Given the numbers -- 0.5 << 375 M -- i don't get which is richer than the other!

- p5 l57-59 "There are also many peaks which are more distinguished ..." distinguished seems unclear to me. Could you clarify?

- p6 l17 "overlapped with SD and repeats". what is SD?

- p7 l7 "yields an order of magnitude more peaks (279 to 411) than MPGs". But 411/279 < 2? which order of magnitude?

- p7 l42 "q-values" of what?

- p10 l31-32 "Graph peak caller [18], a graph MACS2 implementation, by using the same genome size ..." How do you compute the genome size for a genome graph?

* typos

- Fig 1. b: typo in GPG box

**Authors Response**

**Point-by-point responses to the reviewers’ comments:**

*We would like to thank the reviewers for their constructive criticism and valuable comments. We made an effort to incorporate all suggestions and address concerns. We believe that our paper is now improved and our message straightened. See point-by-point responses below.*

Reviewer 1

Referee report for “Personalized and graph genomes reveal missing signal in epigenomic data”

This manuscript evaluates the impact of choice of reference genome for alignment in ChIP-seq analysis. Currently, the standard practice relies on reference human genome for identifying peaks, and modified personal genomes for identifying allele specific occupancy. However, there has not been systematic efforts in investigating the pitfalls of using reference genomes. The paper considers three alternatives to reference genome: modified personal genomes that are obtained by modifying the reference genome by replacing the SNPs, denovo assembled genome, and finally graph personalized genome which provides an alternative between the first two choices. While I appreciate the effort, there are a number of significant weaknesses pertaining the analyses.

1)	The overall conclusion seems to be that using a personalized genome identifies a small number of additional peaks and removes an even smaller number of peaks that were identified by the reference genome. While this is a reasonable observation, given the instability of peak callers, these percentages may not be significant (or they may be in the order the variation one tends to see if the alignment or peak calling parameters are slightly varied).

The manuscript uses MACS for peak calling, but does not specify what level of false discovery rate is used. One issue with MACS and other peak callers is that they cannot reliably control FDR. The ENCODE project developed IDR as an alternative. Carrying out these investigations with IDR would be more robust and conclusive.

*We thank the reviewer for recommending IDR. We note that IDR relies on replicates to produce its results, which might not always be available. Looking in ENCODE, in the cell line we used, the replicates for H3K4me1 (ENCSR000AKF) and H3K27ac (ENCSR000AKC) are sequenced at different read length and very different read depth. Since IDR method is dominated by the weakest replicate, we are reluctant to use these replicates because their discrepancies may hide the altered peak calls. Nevertheless, we did locate a new dataset, ENCODE H3K4me3 (ENCSR057BWO), that has consistent read length and read depth between replicates. We chose to run IDR on this dataset. We ran IDR on peaks called using these data in the reference and personalized genome to obtain two lists of IDR-corrected peaks. Then we overlap the two peak lists as before to obtain the list of altered peaks. The results are summarized in the new Table S6 in the supplements:*

*Peak type	MACS2 (FDR 0.05)	IDR*

*Reference peaks	29484	21764*

*NA12878 GPG peaks	29491	21785*

*Personal-only peaks	293 (0.99%)	138 (0.6%)*

*Reference-only peaks	77 (0.26%)	85 (0.39%)*

*We now summarize these new results in the GPG section of the manuscript:*

*“An additional concern is that the FDR of peak callers such as MACS2 may be inaccurate. Therefore, we tested IDR, a tool that compares the ranking of peaks between replicates to better control the FDR [26]. Using our approach on an additional NA12878 ENCODE dataset (H3K4me3), due to the availability of consistent replicates with similar read depth and read length, we found that the proportion of personal-only peaks in this dataset declines from 0.99% to 0.6% if we correct MACS2 peaks with IDR (Table S6). This shows that the majority of personal-only peaks remain, even if we apply a more stringent statistical cutoff.”*

*We also added to the Methods:*

*“In the H3K4me3 replicate analysis, Graph Peak Caller was run on the alignment of each replicate and also on the merged alignments. IDR [26] was run on the peaks of each replicate to obtain corrected peaks to be compared against the peaks of the merged alignments.”*

*Secondly, to test directly the effect of the choice of FDR, we also varied the FDR level from 0.05 to 0.01 and show that the number of personal-only peaks only decreases by a quarter as the FDR approaches 0.01 in both H3K4me1 and H3K27ac.*

*Fig S11: The number of APs calls increases when relaxing the false discovery rate (FDR) parameter from*

*0.05 to 0.10. Conversely, the number of APs decreases by roughly a quarter as the FDR approaches 0.01. This decrease is mild given that the FDR parameter is five times more stringent.*

*We added these new results in the manuscript and amended the methods to be more explicit about the fact that we used a peak caller FDR level of 0.05. The new text in the GPG result section is as follows:*

*“We also check that the number of personal-only peaks was not too sensitive to the choice of FDR threshold (Fig S11).”*

2)	Another concern is that it is not clear whether the APs (altered peaks) can be recovered by varying the thresholds of peak calling. In other words, perhaps the peaks specific to personalized genomes are just below the peak-calling threshold when the data is analyzed with the reference genome (e.g., they are not identified at 5% FDR but make it to the list in 10%). In this case, APs arise due to change in ranking of the peaks and one would need to argue that the new ranking is somehow more meaningful. Authors should make a clear distinction between APs that will never be recoverable (not even with higher depth) unless one uses personalized genomes. Fig4a depicts such peaks as a smear in the MA plot, but it is not clear whether these are as reproducible as the ones with some reads with the reference analysis.

*As the reviewer correctly points out, it is entirely possible that a shallow read pileup may not be called in the reference but become a peak called in the personalized genome given the same FDR. However, we also anticipate cases like the one shown in Fig 6d where relaxing the FDR should never yield a peak call since the read depth is near 0 in the reference. As suggested, to count the number of such events, we reran peak calling in the reference graph using a relaxed q-value threshold of 0.10 and eliminate the altered peak calls (FDR 0.05) that appear in the new reference peak list (FDR 0.10). We present and interpret this new data in the result section “Further characterizing the altered peaks”:*

*““The false discovery rate (FDR) is an important parameter in peak calling. It is possible that a peak that is personal-only at a given FDR would be found in the reference at higher FDR. Testing for this we found that for H3K27ac, 1021 of 1847 peaks remain personal-only at 0.05 FDR even when using 0.10 FDR in the reference. For H3K4me1, 745 of 3068 peaks remain personal-only. This suggest that personal genomes move some read pileups just above the significance threshold. We know that personalized genomes improve transcript abundance estimates in RNA-seq [11]. Provided the signal also increases in ChIP-seq, we argue that the peak ranking in personalized genomes is more meaningful than the ranking in the reference genome. The excess of personal-only to ref-only peaks supports the new ranking. If the ranking changed randomly, the number of personal-only peaks would equal ref-only peaks.”*

*As for the smeared peaks, we must note that these peaks are mostly reference-only peaks that are found with the DPG approach. They are very rare in MPGs and GPGs. Such peaks should probably be treated as an artifact since they are produced by missing regions in the denovo assembly that probably exist in the real genome, but failed to be assembled. Therefore, even if smeared peaks happen to be more reproducible, these peaks are less interesting since they are not the product of genetic variation.*

*We add this point to the DPG result section:*

*“We view these ref-only peaks as probably missing from the denovo assembly and not as the product of genetic variation.”*

3)	A related issue is also the impact of alignment on peak calling. It is important to show that the variation observed with the change of alignment genome is larger than that would arise by changing the alignment parameters.

*The reviewer suggests an important analysis. To test whether using a personal genome has more impact on peaks than changing the alignment parameters, we ran several sets of parameters varying the cost of SNPs and gaps when aligning to the reference and peak calling. Next, we counted altered peak calls relative to the default aligner parameters. We find that the number of altered peaks produced in this manner is generally lower than that produced by personalized genomes. The results are summarized in the new supplemental figure S12 and in the GPG result section:*

*Fig S12b,c: b) Number of mismatch and gap events per read with respect to alignment mismatch and gap penalties. Relaxing penalties when aligning to the reference*

*admits many poor quality alignments. c) Number of altered peaks created by aligning reads to the reference genome and varying gap and mismatch penalties in H3K4me1 (top) and H3K27ac (bottom). The lowest penalties can generate more peaks than a personalized genome by admitting many poor quality alignments that would be rejected with default parameters (especially in the H3K27ac dataset).*

*Personal genomes generate thousands of APs without admitting poor quality alignments.*

*“Moreover, since alignment parameters may also be relevant, we confirm that simply aligning ChIP-seq reads to the reference with different mismatch and gap penalties does not change more reference peak calls than a personalized genome (Fig S12b, S12c).”*

4)	It is not clear how accurate the alignments are with GPGs or MPGs compared to reference genomes? A computational experiment for quantifying this is warranted.

*The reviewer is right to be concerned with the gains in alignment accuracy brought by personal and graph genomes. Their concern is shared with groups that implement and publish graph genome alignment tools. Therefore, this result is often published together with the alignment tools (vg map). For instance, Figure 2a from h ttps://doi.org/10.1038/nbt.4227 shows that graph population genomes increase the alignment accuracy over the reference. We think that this result provides an excellent comparison of alignment accuracy between personalized genomes and reference genomes. Because the focus of our study is on altered peaks, we didn’t want to expand too much on the quality of read mapping, but we have now added a statement on improved alignment accuracy in genome graphs in the introduction and cited the above paper. In addition, we have also added a reference mentioned by Reviewer 2, which details the impact of graphs on read mapping: doi:10.1186/s12859-016-1103-9. For modified reference genomes, similar improvements are reported in Fig 7 of doi:10.1186/s12864-019-5854-3.*

5)	It is also important to investigate whether APs specific to personalized genomes are more supported by external data (ATAC-seq, RNA-seq or some other) compared to lost APs (reference genome specific peaks).

*The reviewer points out that robust altered peaks should be supported by orthogonal sources of data. To this end, we note that histone marks are often correlated with other histone marks as a consequence of the epigenetic code. Therefore, we conducted an additional analysis to check for additional support of altered peaks by correlating them with other histone marks. We show that read depth in the altered peaks of one mark increases on average with the read depth in other marks similarly between all peaks and the altered peak calls (see Fig S13).*

Fig S13: Average H3K4me1, H3K27ac and H3K4me3 read depth of common, personal-only and ref-only peak calls in the NA12878 GPG. Histone peaks show correlation patterns characteristic to the histone code. Personal-only calls show an increased read depth compared to ref-only calls.

*We present this result in the GPG result section:*

*“We also show that personal-only peak are supported by orthogonal sources of data in addition to replicates. We do so by correlating the peak calls to the read depth in H3K4me1, H3K27ac and H3K4me3. In common peaks, the average read depth rises together with the average read depth of correlated histone marks (Fig S13). In personal-only peaks, we observe a similar pattern together with an increased average read depth compared to ref-only peaks.”*

*We added to the Methods:*

*“The histone mark correlations were generated with HOMER [50] to support altered peaks with orthogonal data.”*

6)	What is the impact of personalized genomes on allele specific quantification? Are the authors seeing that some of the APs specific to personalized genomes would not have been detected by allele-specific quantification.

*The reviewer asks a good question since some allelic specific quantification methods make use of personalized genomes. For example, AlleleSeq generates MPGs to assign reads to the best allele. In our results, we see common peaks with significantly changed read counts. We expect that some of these should be picked up by allelic specific quantification methods. However, a factor leading to altered peaks is unmapped reads that are rescued by the personalized genome (ex: Fig 6d). We do not expect allelic specific quantification methods to recover such peaks, unless they too remap all the reads to a personalized genome. Unfortunately, as far as we’re aware, no allelic quantification pipeline for graph genomes is currently available. Furthermore, we emphasize that personalized graph genomes aid allelic specific quantification since they can map reads directly to all known alleles in an individual or population. The increased mapping accuracy could also lead to better estimates of ChIP-seq fragment counts.*

*We have added this remark when discussing Fig 6d:*

*“Since this peak lies on the alternate allele, future allelic quantification pipelines that operate on graph genomes should be able to detect such events.”*

Reviewer 2

*	Summary

The authors investigate whether a graph representing a reference genome and known variations helps calling ChIP-seq peaks compared to when the analysis is performed against a single reference genome.

This study belongs to a group of studies that seek to delineate the improvements brought by using genome graphs. It is a natural question since the sequencing of multiple individuals brings key knowledge on individual variations. Beyond the single linear reference genome (which is the current gold standard for read analyses), three alternative personalized graphs are considered: modified personalized genome (MPGs), graph genomes (GPG), and de novo assembled personal genomes (DPGs). In this order, each graph incorporates more variability, and provides higher fidelity to the individual genome.

*	Pedagogical aspects

The use of genome graphs is not common place and may puzzle a lot of readers.

A broader general introduction about this concept, and its variants would help the readers to grasp the potential impact of genome graphs, which will likely become standard within 5-10 years from now (when available of course).

Before entering the results section, please provide a clear definition (at least description) of each type of genome graphs, and explain their differences, which could be illustrated with well chosen examples. For instance, Figure 1 does not illustrate the fact that you use phased variants for creating the MPG. The legend should be more explicit regarding the strike G in the MPG box. Moreover, a pointer to a recent review:

Computational Pan-Genomics: Status, Promises and Challenges

Briefings in Bioinformatics, Vol 19(1), p. 118-135, doi: 10.1093/bib/bbw089 2018

would complete these explanations and allow the readers to extend their understanding of the topic.

*We thank the reviewer for the suggestion and the pointer to a relevant paper. We agree that genome graphs remain mysterious for most people. We have now extended the introduction to provide a brief explanation of genome graphs and to include example applications beyond our own, as enumerated in the paper provided above. We have also modified Figure 1 to illustrate two adjacent SNPs and their phasing in the modified reference, genome graph and phased denovo assemblies. We also added a few more descriptive statements in the introduction of each genome implementation:*

*“Here, we employ a phased denovo assembly of NA12878. Like MPGs, it provides a sequence for each haploid but it is not constructed from the reference genome.”*

*“Finally, the above trade-offs also motivate the exploration of graph genomes as an additional strategy. Graph genomes are a flexible way of representing many possible sequences in a concise data structure [15]. Unlike traditional one dimensional sequence representations, graph genomes split sequences into segments called nodes. The nodes are connected to each other by edges, which allows traversing the graph from one node to another. Well-defined rules about the semantics of nodes and the direction of edges allow graphs to express many sequences. A valid traversal is called a path and represents one possible sequence that is represented by the graph. This emerging technology can encode sequence variation at many levels for different purposes [16]. For example, it can encode genetic variation within a population of the same species, genomic differences between species within a phylogenetic tree or genomic rearrangements of a cancerous tumour.*

*We employ graph genomes to construct a graph personalized genome (GPG) representing the diploid genome of a single individual (Fig 1b). GPGs can leverage available call sets that include a broad range of variants, from SNPs and indels to catalogues of sequence resolved SVs and also capture the diploid nature of the human genome [17]. This is achieved by converting the reference genome to a graph format and augmenting it with nodes representing the variants. By mapping to a GPG, we expect that reads containing variants will align to the appropriate path, which improves read alignment accuracy [5].“*

*	General major remarks.

1.	The peak calling is evaluated with two distinct epigenetic marks. Can you give a rationale behind this choice? Why do you consider only two? Could you justify that two is enough for this study, which seeks to yield general conclusions? In any case putting some background knowledge about each mark in your introduction can help the reader grasping the context.

*It’s true that the rationale for the selection of these two histone marks was not explicit in the first version of the manuscript. We now mention in the introduction that the epigenetic marks were chosen to match those available in the Blueprint dataset, which we wanted to use for comparison in actual tissue samples (e.g in Fig 3). Despite using only two marks, we still managed to extract relationships from our results that can be generalized to other ChIP-seq datasets. For example, our logistic regression model shows that larger peaks are less likely to be altered. Therefore, we can expect fewer altered peaks in broad histone marks and more altered peaks in narrow histone marks and transcription factors. We have now added the explanations and also added a brief description of the two histone marks in the introduction to help readers grasp the context.*

*“We focus on the H3K4me1 and H3K27ac histone marks primarily due to broad availability in samples of the Blueprint Consortium [20] we intend (see below).*

*“This model predicts fewer altered peaks in broad histone marks and more altered peaks in narrow histone marks and transcription factors.”*

*“H3K27ac is linked with enhancers, distinguishing between active and inactive enhancers and therefore impacts gene regulation [21]. H3K4me1 correlates with H3K27ac in enhancers, but can interact with chromatin regulators such as p300 and other histone marks to determine other classes of regulatory elements [22]. Even if only a fraction of peaks are observed to be altered, these regions will correspond to biochemically active regions that are more likely to differ between individuals and, as such, could be relevant in the study of various human phenotypes.”*

2.	Species Similarly, this world concentrates on Human data, which may be justified by the quality of reference and the availability of high quality known variations.

*The reviewer is correct in this observation. We now include a remark in the discussion emphasizing the importance of genetic diversity in reference bias:*

*“However, the above results could be amplified in species of greater genetic diversity such as chimps [31].”*

3.	In my view, most of the results follows from the difference in read mapping, rather than in peak calling. It has already been shown that read mapping against a sequence graph rather than against a linear reference sequence, strongly impacts read mapping (see e.g. BMC Bioinformatics

doi:10.1186/s12859-016-1103-9, 2016, and later works citing this one). I would find valuable that you detail the read mapping results against the three graphs and evaluate the impact of read mapping parameters on these, before investigating the peak calling impact. It is necessary to understand the obtained results.

*We agree with the reviewer that a sufficient number of read mapping differences must occur before a peak call is changed. Some of these results were already presented in supplementary tables S4a, S4b and S4c, which showed a detailed breakdown of read mapping differences in MPGs, DPGs and GPGs relative to the reference genome. In these tables, we see that the proportion of altered peaks increases with the proportion of changed read mappings. For instance, MPGs have the fewest number of altered peaks only change the mapping of 3% of reads. GPGs register an intermediate number of altered peaks and change the mapping of 8% of reads. This increase over MPGs is likely due to representing the full diploid genome. Following this trend, DPGs register the largest number of altered peaks as well as the larger number of changed mappings (9%).*

*However, as the reviewer correctly points out, we don’t know how the choice of alignment parameters may change these values. Therefore, following a suggestion that was also made by Reviewer 1, we show new results in which we independently change the mismatch and gap penalties (see above and Fig S12a).*

*Fig S12a: a) Impact of the gap and mismatch alignment penalties on the proportion of changed mapping between the reference and the NA12878 GPG. This proportion only falls by 1% even when using the most lenient alignment penalties.*

*We have now added in the GPG result section:*

*“We verified that this proportion remains stable when varying alignment mismatch and gap penalties (Fig S12a).”*

Your observation from p 5 l 17 and beyond "We found, as expected, that as the read length increases, APs become less likely (Fig 3d). We repeated the NA12878 WGS alignment comparison with the longer 100bp reads to gain some insight on why this happens (Table S4a). Compared to the shorter reads (Table S1a), the longer reads show a proportionally small decrease in aligned reads with unequal mappings." really supports the view that mapping is the key step in the whole analysis in terms of impact.

Other observations later p7 l11-12 suggest that testing mappers that can better handle indels could change the results. Could you consider such mappers (e.g. GMAP) in your tests?

*As demonstrated by our Figure 1 and 2 and by our logistic regression analysis, the reviewer is correct to note that indels play a major role in the creation of altered peaks. Due to the novelty of graph genomes, aligners such as GMAP are not available for graph genomes. Our understanding is that GMAP was optimized for RNA-seq reads. While this could handle indels better, it might also have other unintended consequences on ChIP-seq reads. We also want to emphasize that our contribution is not in showing improved alignment in personalized genomes, but to detail their downstream impact on peak calling. This is why we chose to use bwa mem and vg map aligners for ChIP-seq data. Nevertheless, following suggestions we have added analyses showing the impact of varying the gap penalties within vg map (see new Fig S12). We showed that the proportion of changed mappings never falls below 7% even with the most lenient gap penalty and plateaus at 8% with the most stringent gap penalties. We have added this point to the discussion as it will become relevant once other graph aligners become available.*

*“ Comparing the results of several graph genome aligners that employ different strategies in addition to varying alignment parameters would be interesting when they become widely available.”*

4.	Beyond mapping, the peak calling parameters may impact the results. Could you please investigate further the robustness of your results with respect to major peak calling parameters?

*See above for the answer to a similar question from reviewer #1 (which includes a description of new Fig S12 and Table S6).*

5.	In your description, the DPG is the ultimate graph solution in terms of genome fidelity. Maybe this is not completely true. This view disregards the variations at organs or cell levels. What about the pan-cellular graphs built from multiple single cell whole genome samples? Similarly, to extend MPGs, how could you incorporate the relative frequency of known variations in your graph to improve predictions?

*We agree with the reviewer and recognize the value of this suggestion, especially in the context of single cell multiomic sequencing methods. We have added these points in our discussion:*

*“Finally, we constructed personalized genomes that only incorporate germline genetic variation. However, this does not account for somatic genetic variation that is known to exist at the cell and tissue level [32]. The construction of pan-cellular genome graphs is another direction to explore, especially as single cell multiomic technologies matures [33].”*

*As for the second point, the population frequency of alleles could be used to create a population level MPG. For instance, we would replace the reference allele with the major allele found in the population. In this paper however, we focused on variation at the individual level, which does not require knowledge of allele frequencies.*

6.	Reproducibility

I have not attempted to reproduce your results. The description provided in the Methods section give a broad view of your computational experiments. However it is not sufficient for full reproducibility. Could you provide in supplementary material a Snakemake script (or several) that copes with software installation and can be run to fully reproduce your quantitative results and figures?

*We would be happy to see other groups reproduce our results. We have now clean-up our code and published a GitHub repository containing several pipelines under the “Code release” subsection in Methods. We include a nextflow pipeline to generate the genomes graphs from a VCF file and another nextflow pipeline that generates altered peaks from genome graphs and ChIP-seq reads. This code is portable and should run on any HPC with vg and Graph Peak Caller installed. Additionally, we include perl and bash scripts to generate altered peaks from MPGs. Lastly, we include another perl script used to generate the chain files required to lift annotations from the denovo assembly to the reference genome. Link to github repository:*

*h ttps://github.com/cgroza/personalized_genomes_gbio We have added a subsection to the Methods:*

*“Code release*

*We release code that builds the personal genomes, aligns ChIP-seq reads and calls altered peaks at: https://github.com/cgroza/personalized_genomes_gbio”*

7.	Do your findings have biological implications?

Sentences like p8 - l8. You support your claim with one example. In my view, it is insufficient. I am persuaded that sequence graphs makes a difference compared to a single reference genome, but I have the impression that your results do not suggest this difference have a critical impact on biological considerations. This is a difficult question. Looking at differences e.g. in enhancer regions that would impact transcription initiation rate, could help showing that ChIP-seq peak calling is improved thanks to genome graphs.

*We agree with the reviewer that demonstrating critical biological impact would be great. We included the GWAS example as a potentially interesting incidental finding. Moreover, we added the genomic distribution of altered peaks relative to genes to verify that they follow the same patterns as the common peaks, and not necessarily for biological impact. Since this work was conducted on a cell line, selected because of its high-quality genome to be used as a benchmark, it is inherently limited in showing biological significance.*

*To address this point, we made the following edit in the GPG section:*

*“Such examples justify investigating whether GPGs could improve our understanding of gene regulation in individual genomes.”*

*We elaborate in the discussion on how we may evaluate the biological impact of our analysis:*

*“Notably, to properly determine the biological significance of APs, our analysis will need to be expanded to datasets obtained from more tissues and with follow-up experiments.”*

*	Precise remarks

-	p2 l18 (MPG) is singular We corrected the typo.

-	p3 l54 "Ideally, peaks that have AP calls should also have a skewed coverage." Could you be more precise and explain why it'd be ideal?

*We have modified this sentence to say: “that indicates a clear change in read depth at that site.”*

-	p3 results: clarify the notion of personal-only, ref-only and common peaks, as well as Altered peaks, given that peaks may inexactly overlap, may get similar, but still unequal coverage.

*We have added brief definitions for personal-only, ref-only and common peaks in the results section and point to the Methods section. We also edited the Methods section for details regarding coverage differences and inexact overlap between peaks.*

- p3 l59: 30 out of ??

*We have modified the text to say 30 out of 1600.*

-	p4 l7 define "lower confidence"? Explain how you compare peaks' confidence

*Here we use the MACS2 score as a measure of confidence, which is just a linearly scaled q-value. We clarify this in the manuscript. We have qualified the statement with “(as measured by MACS2 score)”.*

-	p4 l52-53 "as the NA12878 variant call set (3.5M SNPs, 0.5M indels) is richer than Blueprint (approximately 3.25M SNPs and 375M indels per sample, Fig S4c)." Given the numbers -- 0.5 << 375 M

-- i don't get which is richer than the other!

*Thank you for catching this. There was a missing leading zero in front on 375M. The NA12878 variant set is richer. We corrected this typo.*

-	p5 l57-59 "There are also many peaks which are more distinguished ..." distinguished seems unclear to me. Could you clarify?

*Rephrased “more distinguished” as larger read count difference between personal and reference read count.*

-	p6 l17 "overlapped with SD and repeats". what is SD?

*We are not explicitly mentioning “segmental duplications (SD)”.*

-	p7 l7 "yields an order of magnitude more peaks (279 to 411) than MPGs". But 411/279 < 2? which order of magnitude?

*That is not the comparison we intended to make here. We intended to compare with the number found in MPGs. We rephrased the sentence to be less ambiguous.*

-	p7 l42 "q-values" of what? We mean the q-values of peak calls as found in the MACS2 and Graph Peak Caller output. We edit to say “q-values of peaks”.

-	p10 l31-32 "Graph peak caller [18], a graph MACS2 implementation, by using the same genome size ..." How do you compute the genome size for a genome graph?

*Its true that each path through a genome graph will have a different length. But they should all be very close to the linear reference genome. We use the same genome size as the linear genome size used in MACS2.*

*	typos

-	Fig 1. b: typo in GPG box *We corrected the typo.*

**Second round of review**

**Reviewer 1**

I commend the authors for the additional computational experiments that they have performed. The newly added data will be useful towards changing the practices of analyzing ChIP-seq data in epigenomewide studies.

I disagree on the interpretation of some of the results and have the following concerns.

- Using IDR, which is the community standard for a rigorous analysis, leads to almost 40% reduction in the number of identified personal-only peaks. This is significant and should be commented on as such.

In addition, ENCODE has so many ChIP-seq datasets -- many TF ChIP-seq from GM12878 (and some TF-GM12878 combinations from multiple labs) in addition to histones -- and they have all gone through QC so the authors could choose high quality datasets to quantify the gains due to GPGs. I would definitely like to see a more robust number on the potential percentage of APs that will emerge with MPGs- showcasing only in one dataset will be significantly short of GB standards. This is especially important because, as the authors point out, we will likely have one replicate of a epigenomic mark (as in blueprint) from an individual so peak calling will need to rely on more stringent thresholds not to inflate false positives.

- For the blueprint analysis, a convincing argument could be made by quantifying the # of APs in terms of how much each is shared among different subjects and whether the overlap among subjects can be explained by the SNP alleles and other SVs shared by the subjects at the AP location. I think this would add a lot value to the analysis and could help to make the case that for epigenomewide association analysis, peak peaks should be made by MPGs.

**Reviewer 2**

Review on the revised article:

Personalized and graph genomes reveal missing signal in epigenomic data

By Groza et al.

I'd like to thank the authors for considering the reviewers comments and for the improvement of their manuscript.

Apart for the minimal changes i suggest below, i think that the article is worth publishing in Genome Biology and could thus be accepted.

* Read mapping and Alignment parameters

The new additional figures and evaluation that were made for this revision suggest that the impact of read mapping or alignment is important for the improvement of peak calling you obtained using PG or Graph genomes.

There are several interesting evidence:

1. The impact on APs of alignment parameters (gap/mismatch penalties) differs between the two marks. It supports the idea that the improvement brought by PG is variable with the epigenetic mark. Hence, to apply this method one may have to tailor it somehow for the marks one targets.

2. GWAS example (Fig 6c and 6d): It is nice and interesting to see this example where a lot of SNVs are incorportated in the graph genome, which allows aligning more reads in that region. It suggests that genomic regions with higher density (relative to the average density over the whole genome) of SNVs are likely to exhibit the most improvement. Together with your findings on regions that cumulates small indels and SNVs, it supports the idea that improvements occur in genomic region where read mapping/alignment is more difficult.

Altogether, the "upstream" benefit of the improved read mapping/alignment due to PG/graph genomes, should be stated explicitely in your conclusion or discussion. By upstream, i mean upstream in the pipeline compared to the peak calling step. Could you consider adding one sentence or two about this?

* Not rephrased ?

%%%%%%%%%

- p7 l7 "yields an order of magnitude more peaks (279 to 411) than MPGs". But 411/279 < 2? which order of magnitude?"

That is not the comparison we intended to make here. We intended to compare with the number found in

MPGs. We rephrased the sentence to be less ambiguous.

%%%%%%%%%

The sentence has remained the same and was not rephrased. It is still confusing.

* Typos

- Table S4 b: typo in legend "is 9.43%%."

- Discussion: l 399: "multiomic technologies matures": mature without S

- p9 l 219 denovo -> de novo

**Authors Response**

**Point-by-point responses to the reviewers’ comments:**

Reviewer #1:

I commend the authors for the additional computational experiments that they have performed. The newly added data will be useful towards changing the practices of analyzing ChIP-seq data in epigenomewide studies.

I disagree on the interpretation of some of the results and have the following concerns.

- Using IDR, which is the community standard for a rigorous analysis, leads to almost 40% reduction in the number of identified personal-only peaks. This is significant and should be commented on as such.

*We edited the abstract to further emphasize the relaxed cutoffs used for the initial estimates (which still correspond to what is often used for such analyses):*

*“Using permissive cutoffs, MPGs are found to alter approximately 1% of peak calls while DPGs alter up to 5% of peaks.”*

*We also added a reminder in the discussion about the reduction in altered peaks after correcting with IDR:*

*“The proportions of altered peaks in GPGs only decreased by a factor of two in the stringent IDR analysis that employs ENCODE replicates.”*

- In addition, ENCODE has so many ChIP-seq datasets -- many TF ChIP-seq from GM12878 (and some TF-GM12878 combinations from multiple labs) in addition to histones -- and they have all gone through QC so the authors could choose high quality datasets to quantify the gains due to GPGs. I would definitely like to see a more robust number on the potential percentage of APs that will emerge with MPGs- showcasing only in one dataset will be significantly short of GB standards. This is especially important because, as the authors point out, we will likely have one replicate of a epigenomic mark (as in blueprint) from an individual so peak calling will need to rely on more stringent thresholds not to inflate false positives.

*Showing results for a wider array of ChIP-seq datasets would indeed inform user’s expectations.*

*We have sourced the majority of the ENCODE Reference Epigenome for GM12878*

*(E	NCSR447YYN) which includes other histone marks (H3K36me3, H3K9me3), POLR2A, EP300, and a transcription factor (CTCF) and ran our GPG pipeline. Below we include a new figure with a caption explaining the new result:*

*Figure S13: Proportion of personal-only and ref-only peaks generated by different NA12878 ENCODE dataset. Top and bottom annotations show the numbers of APs and total peaks respectively. Histone marks, POLR2A and EP300 show similar or higher proportions of altered peaks while the transcription factor CTCF shows the lowest impact. H3K27me3 shows an unusually small number of total peaks, which may explain a larger than usual proportion of APs, especially for ref-only peaks.*

*We introduce the above figure in the GPG section of the paper:*

*“We ran even more ENCODE datasets through our GPG pipeline to measure the proportion of APs across the NA12878 reference epigenome (Fig S13). GPG-only peaks were found to vary from 0.84% for CTCF to 6.53% for H3K9me3.“*

*ENCODE NA1278 Reference Epigenome:*

*h ttps://www.encodeproject.org/reference-epigenomes/ENCSR447YYN/*

-	For the blueprint analysis, a convincing argument could be made by quantifying the # of APs in terms of how much each is shared among different subjects and whether the overlap among subjects can be explained by the SNP alleles and other SVs shared by the subjects at the AP location. I think this would add a lot value to the analysis and could help to make the case that for epigenomewide association analysis, peak peaks should be made by MPGs.

*We agree that associating genotypes to altered peaks would indeed be very informative. However, this analysis would best be performed by constructing a large pangenome graph that represents all the genetic variation in Blueprint samples, together with all the Blueprint haplotypes. Then we could look for both genotype and haplotype associations with altered peaks without having to construct hundreds of personalized genomes. So far, this work only focused on diploid genome graphs. Therefore, we reserve this analysis for future work*

*We edited the discussion to address this potential new analysis:*

*“Furthermore, as pangenome graphs are created to capture all known variations [15] together with the haplotypes of entire populations, it might be possible to further improve on the current performance as well as finding associations between genotype and personal-only peaks.”*

Reviewer #2

I'd like to thank the authors for considering the reviewers comments and for the improvement of their manuscript.

Apart for the minimal changes i suggest below, i think that the article is worth publishing in Genome Biology and could thus be accepted.

* Read mapping and Alignment parameters

The new additional figures and evaluation that were made for this revision suggest that the impact of read mapping or alignment is important for the improvement of peak calling you obtained using PG or Graph genomes.

There are several interesting evidence:

1.	The impact on APs of alignment parameters (gap/mismatch penalties) differs between the two marks. It supports the idea that the improvement brought by PG is variable with the epigenetic mark. Hence, to apply this method one may have to tailor it somehow for the marks one targets.

*We have shown additional evidence on this topic in the new Fig S13, showing the impact of our GPG pipeline over all ChIP-seq datasets in the NA12878 ENCODE reference epigenome.*

2.	GWAS example (Fig 6c and 6d): It is nice and interesting to see this example where a lot of SNVs are incorportated in the graph genome, which allows aligning more reads in that region. It suggests that genomic regions with higher density (relative to the average density over the whole genome) of SNVs are likely to exhibit the most improvement. Together with your findings on regions that cumulates small indels and SNVs, it supports the idea that improvements occur in genomic region where read mapping/alignment is more difficult.

Altogether, the "upstream" benefit of the improved read mapping/alignment due to PG/graph genomes, should be stated explicitely in your conclusion or discussion. By upstream, i mean upstream in the pipeline compared to the peak calling step. Could you consider adding one sentence or two about this?

*We have added a new statement to make this point explicit in the first paragraph of the discussion:*

*“These personalized genomes provide an upstream benefit by improving read alignment to downstream ChIP-seq peak calling pipelines.”*

* Not rephrased ?

-	p7 l7 "yields an order of magnitude more peaks (279 to 411) than MPGs". But 411/279 < 2? which order of magnitude?"

That is not the comparison we intended to make here. We intended to compare with the number found in

MPGs. We rephrased the sentence to be less ambiguous.

The sentence has remained the same and was not rephrased. It is still confusing.

*We have rearranged this sentence to emphasize that 297 to 411 is a range of skewed peaks for GPGs and not MPGs:*

*“The same test for read count skew yields between 279 and 411 peaks, an order of magnitude more than MPGs.”*

* Typos

-	Table S4 b: typo in legend "is 9.43%%." - Discussion: l 399: "multiomic technologies matures": mature without S

-	p9 l 219 denovo -> de novo

*We have removed the typos and made another attempt at rephrasing the ambiguous sentence. Thank you for your close attention*
